# A novel mechanism of action of HER2 targeted immunotherapy is explained by inhibition of NRF2 function in ovarian cancer cells

**DOI:** 10.18632/oncotarget.12425

**Published:** 2016-10-04

**Authors:** Hilal S. Khalil, Simon P. Langdon, Alexey Goltsov, Tero Soininen, David J. Harrison, James Bown, Yusuf Y. Deeni

**Affiliations:** ^1^ Division of Science, School of Science, Engineering and Technology, Abertay University, Dundee, DD1 1HG, United Kingdom; ^2^ Division of Computing and Mathematics, School of Arts, Media, and Computer Games, Abertay University, Dundee, DD1 1HG, United Kingdom; ^3^ Division of Pathology, Institute of Genetics and Molecular Medicine, University of Edinburgh, Western General Hospital, Edinburgh, EH4 2XU, United Kingdom; ^4^ School of Medicine, University of St Andrews, St Andrews, KY16 9TF, United Kingdom

**Keywords:** Pertuzumab/Trastuzumab, NRF2, ROS, HER2-targeted, cancer-immunotherapeutics

## Abstract

Nuclear erythroid related factor-2 (NRF2) is known to promote cancer therapeutic detoxification and crosstalk with growth promoting pathways. HER2 receptor tyrosine kinase is frequently overexpressed in cancers leading to uncontrolled receptor activation and signaling. A combination of HER2 targeting monoclonal antibodies shows greater anticancer efficacy than the single targeting antibodies, however, its mechanism of action is largely unclear. Here we report novel actions of anti-HER2 drugs, Trastuzumab and Pertuzumab, involving NRF2.

HER2 targeting by antibodies inhibited growth in association with persistent generation of reactive oxygen species (ROS), glutathione (GSH) depletion, reduction in NRF2 levels and inhibition of NRF2 function in ovarian cancer cell lines. The combination of antibodies produced more potent effects than single antibody alone; downregulated NRF2 substrates by repressing the Antioxidant Response (AR) pathway with concomitant transcriptional inhibition of *NRF2*. We showed the antibody combination produced increased methylation at the *NRF2* promoter consistent with repression of NRF2 antioxidant function, as HDAC and methylation inhibitors reversed such produced transcriptional effects. These findings demonstrate a novel mechanism and role for NRF2 in mediating the response of cancer cells to the combination of Trastuzumab and Pertuzumab and reinforce the importance of NRF2 in drug resistance and as a key anticancer target.

## INTRODUCTION

Nuclear erythroid related factor-2 (NRF2) is a leucine zipper transcription factor and the master regulator of the antioxidant response (AR) pathway. It drives both basal and oxidative stress-induced transcription of a battery of phase I, II, and III detoxification enzymes and cytoprotective genes [1–3], as well as other genes of the metabolic and signal transduction pathways [2–4]. This is achieved by its binding to *cis*-acting factors called Antioxidant Response Elements (AREs) or electrophile response elements (EpREs) within the promoters of its target genes [5]. Under basal conditions, a low level of free NRF2 is available in the cytoplasm where some translocates into the nucleus to drive the basal transcription of target genes. Much of the remaining cytosolic NRF2 is held by Kelch-like ECH-associated protein 1 (KEAP1), a cytoplasmic NRF2-binding adaptor that primes NRF2 for degradation via 26S proteasome [6, 7]. Following oxidative stress or in the presence of NRF2 activators, a number of cysteine residues within KEAP1 become oxidized causing conformational changes in the KEAP1 structure. This disables KEAP1 to bind additional NRF2 and cause its degradation leading to nuclear accumulation of NRF2, activation of its transcriptional function to induce transactivation of ARE-containing genes and finally restoration of cellular redox homeostasis [8].

Hyperactivation of NRF2 is also a recognized intermediate in cellular proliferation and in conferring therapeutic resistance to cancers [9–11]. Specifically, NRF2 activation and KEAP1 inactivation mutations are harbinger to permanent constitutive activation of the NRF2 dependent AR pathway now frequently observed in cancers [12–15]. Furthermore, several therapeutic strategies such as anticancer radio- and chemo-therapy largely depend on reactive oxygen species (ROS) generation to induce cytotoxicity. Thus, hyper-activation of NRF2 dependent AR pathway would attenuate the potency of such agents. Moreover, NRF2 is directly involved in the regulation of apoptosis and proliferation [16, 17].

The HER receptor network contains four members (HER1, HER2, HER3 and HER4) belonging to the family of Receptor Tyrosine Kinases (RTKs), which are key regulators of cellular proliferation, differentiation and survival [18–21]. Activation of RTKs involves ligand dependent homo- and hetero-dimerization and stimulation of their intrinsic tyrosine kinase activity that leads to the phosphorylation of tyrosine residues in the intracellular domain of these receptors. The phospho-tyrosine residues serve as docking sites to recruit a number of signal adapter proteins containing specific domains called SH2- and PTB. Through these domains, RTK links its activation with different cellular signaling pathways such as PI3K/AKT/mTOR, MAPK, and STAT [22, 23]. The activation kinetics and strength of the HER receptor network depend significantly on the expression levels, which vary across different cells and cancers [24]. Likewise it is these variations in expression combined with their context dependent preferences for dimerization partners and ligand dependent or independent signaling modes that cause complexity in fully elucidating the activation and functioning of the HER receptor family.

The HER2 and HER3 receptors are non-autonomous and possess certain defining features, in that HER2 has auto-kinase activity but no known ligands, whereas HER3 is a pseudo-kinase receptor that lacks tyrosine kinase activity. These features dictate the interaction between the HER2 and HER3 receptors and for forming active heterodimer complexes. Mutation or increased gene copy number leads to hyper-activation or overexpression of HER receptors respectively. These mechanisms lead to receptor auto-phosphorylation and constitutive activation, enabling cells to lose their requirement for ligand and/or hetero-dimerization of receptors, mechanisms which elicit uncontrolled proliferative pathways ultimately converting normal cells into malignant cells [18, 22, 23, 25, 26]. Among the RTK kinase family members, HER2 has gained major importance as an anticancer drug target. This is owing to this receptor frequently being overexpressed in certain cancer types and partners with the HER3 receptor, a combination, which is known to strongly elicit robust growth-promoting pathways [27–33].

HER2 targeting immunotherapeutic agents, comprising of HER2 specific humanized monoclonal antibodies, Pertuzumab and Trastuzumab (Herceptin), have acquired a central position as targeted anticancer modalities and are currently being extensively studied [31, 32]. These monoclonal antibodies frequently have limited effectiveness in combating cancers as tumor cells circumvent the action of the single agents due to the readjustments in co-expression of HER2/HER3 receptors, their ligand binding dynamics or changing preference for the dimerizing partners [25, 34, 35]. Because of such complexity, it is imperative to identify and characterize downstream pathway nodes where RTK signaling converges, in order to identify novel druggable targets to exploit during immunotherapies involving HER2 inhibition.

Recently, a growing body of evidence has implicated coregulatory roles of HER2/HER3, NRF2 and ROS in the promotion of cellular proliferation, increased detoxification potential and therapeutic resistance in cancer cells [36–39]. Specifically, generation of ROS, which is a key regulator of the NRF2 pathway [40], has been demonstrated as a regulator of the HER2/HER3 complex and subsequent activation of their functions [41]. Elucidation of these new mechanisms place ROS at a central position where it might act as a point of convergence of these two cytoprotective pathways. Moreover, studies have also shown direct mechanisms of activation of NRF2 by components of the RTK pathway such as PI3K and MAPK [42, 43], whereas many aspects of RTK signaling are regulated by ROS, whose levels are directly modulated by NRF2 function [44]. We have recently shown that NRF2 regulates the expression of HER2 and HER3 to modulate HER2/HER3 targeted immunotherapy against ovarian cancer cells [45]. We have also shown the ROS-dependent hierarchical addiction and manipulation strategy of these cancer cells to draw proliferative advantage [10, 11]. This led us to hypothesize that inhibition of NRF2 function and concomitant cellular accumulation of ROS are possible mechanistic components and basis of action of HER2-targeted immunotherapy.

In this study, we have elucidated a new mechanism of action of the combination of anticancer immunotherapeutic agents. We demonstrate that combination treatment with HER2 targeting monoclonal antibodies, Pertuzumab and Trastuzumab, causes inhibition of NRF2 function and subsequent repression of NRF2 dependent antioxidant response pathway in human ovarian cancer cell lines. This repressive action on NRF2 not only defined the overall sensitivity towards targeted therapy, but could also be modulated to further enhance this sensitivity in otherwise resistant ovarian cancer cells. Furthermore, we present evidence and describe an epigenetic mechanism of transcriptional repression of NRF2, which involves promoter methylation and gene silencing following combination of Pertuzumab and Trastuzumab treatment. Thus we reveal that the effectiveness and enhanced cytotoxic action of the combination of HER2 targeted immunotherapeutic agents can be at least partly explained by their ability to cause transcriptional inhibition of NRF2 and greater repression of its antioxidant function in low, moderate and high HER2 expressing ovarian cancer cell lines. As such, this study expands the role of NRF2 as a key element in driving drug resistance and offers a novel strategy of cancer cell sensitization during the course of targeted therapy for cancer employing immunotherapeutics.

## RESULTS

### Action of targeted immunotherapeutic agents involves generation of ROS, which contributes to cancer growth inhibition

Reactive oxygen species (ROS), while traditionally considered as cellular by-products of metabolism, have gained enormous importance in the past decade and are further recognized as second messengers in signal transduction processes influencing growth, survival and overall physiological homeostasis [46–48]. Furthermore, there are several previous studies that have illustrated the co-modulatory role and interaction of ROS with RTK receptors and growth promoting pathways [49–52]. These taken together with our recent work (10, 11, 45) have led us to hypothesize that inhibition of NRF2 function and concomitant cellular accumulation of ROS are possible mechanistic components and basis of action of HER2-targeted immunotherapy. To address this hypothesis we first studied total ROS levels in basal, Heregulin (HRG) stimulated and drug-inhibited states in three cell lines. HRG is a growth factor belonging to the EGF family. We used HRG as it is a potent ligand for HER receptors and causes HER2-HER3 dimerization [20]. We used a low HER2 expressing ovarian cancer cell line, OVCAR4, a moderately HER2 expressing PEO4 and a HER2 overexpressing cell line, SKOV3 [34].

**Figure 1:**
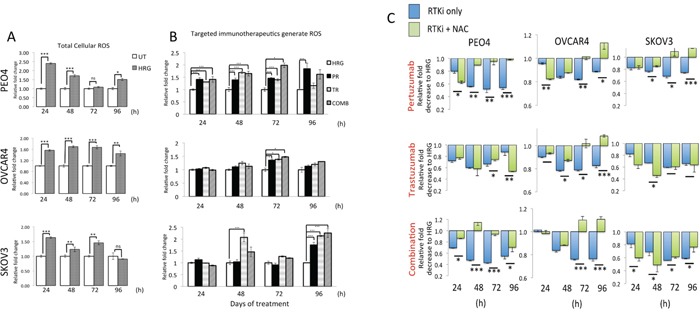
Treatment with HER2 targeting antibodies generates Reactive Oxygen Species (ROS) that when neutralized leads to cytoprotection in ovarian cancer cells **(A)** Heregulin treatment causes persistent elevation of ROS in ovarian cancer cells. Exponentially growing cells were seeded in triplicates in opaque flat bottom black walled 96-well plates for 24 h. Following this, cells were either left untreated (UT) or treated with 1nM Heregulin for different time points as indicated. Following incubations, cells were loaded with DCFDA fluorescent stain for 45 min and assayed for ROS as described in Materials and Methods. **(B)** HER2 targeting monoclonal antibodies cause ROS generation. Cells were seeded as in (A) and treated with either 1nM HRG alone or with co-treatment of 20μg/mL Pertuzumab (PR), Trastuzumab (TR) or their combination (COMB) for different time points as indicated and ROS assay was repeated. For both (A) and (B), the fluorescence reading recorded from each well was normalized to total cell abundance within the same wells as described in Materials and Methods. **(C)** Treatment with N-Acetyl Cysteine (NAC) desensitizes ovarian cells to immunotherapeutic agents targeting HER2. Cells were seeded as in (A) and either left untreated in media containing 1nM Heregulin (HRG) or treated with same media containing 20μg/mL of HER2 inhibitors, Pertuzumab and Trastuzumab either alone or in combination (RTKi only group) or in the continuous presence of 10mM NAC (RTKi + NAC group). Cells were treated with the MTT reagent as described in Materials and Methods and OD readings were obtained. Data shown are mean values ± S.D of triplicates, normalized to UT in (A) or HRG in (B) and (C) and expressed as fold change. Statistical significance was determined between treatment groups either by independent *t* test or ONE WAY ANOVA followed by post hoc Tukey's test as appropriate and significance expressed according to the scale * P <0.05, **P <0.01, ***P <0.001.

Data in Figure 1A illustrate that HRG stimulation alone led to a significant and sustained increase in ROS levels in all three cell lines as compared to basal levels in unstimulated cells. More importantly, we saw that treatments with Pertuzumab, Trastuzumab or their combination led to ROS generation in our ovarian cancer cell line models. ROS elevation was seen at all the time points tested but these were consistently significant in PEO4 cells and more restricted in OVCAR4 (Figure 1B). Within single agent treatments, in PEO4, Pertuzumab generated more ROS than Trastuzumab while in OVCAR4 and SKOV3, Trastuzumab consistently generated higher ROS than Pertuzumab alone.

We then further investigated the contribution of ROS generation in the mechanism of cytotoxic action of these immunotherapeutic agents. To address this, we repeated our cytotoxicity experiment but this time co-treated cells with the ROS scavenger, N-acetyl Cysteine (NAC) in order to neutralize ROS and then study its consequences on survival for different time points of treatment. Strikingly, we found that neutralization of ROS in all the cell lines significantly improved survival following drug treatments (Figure 1C), especially at later time point and perhaps following the uptake and channeling of NAC in *de novo* GSH synthesis. Consistent with our previous conclusions, NAC dependent protection was more pronounced and sustained in the PEO4 cell line and with Pertuzumab and combination treatments, whereas for OVCAR4, NAC was more protective following Trastuzumab and combination treatment. Interestingly, NAC treatment of SKOV3 cells exerted limited protection against cytotoxic action of the inhibitors (Figure 1C). These observations are of significance, as they clearly illustrate the role of ROS and hence of the overall antioxidant potential of cancer cells in determining sensitivity to otherwise unrelated immunotherapeutic agents. The fact that receptor inhibition led to generation of ROS (Figure 1B) and that this ROS was a contributing factor in cellular cytotoxicity (Figure 1C) implicated the engagement of antioxidant pathway during drug action. Thus, we next sought to investigate the status of the NRF2-KEAP1antioxidant response of these cancer cells following the HER2/HER3 targeted immunotherapies. In order to further support and confirm this role, we performed additional experiments as described below.

### Inhibition of NRF2 by Retinoic acid (RA) disrupts its antioxidant transcriptional program, suppresses NRF2 and HO-1 protein levels, elevates cellular ROS and enhances cytotoxicity of the immunotherapeutic agents

**Figure 2:**
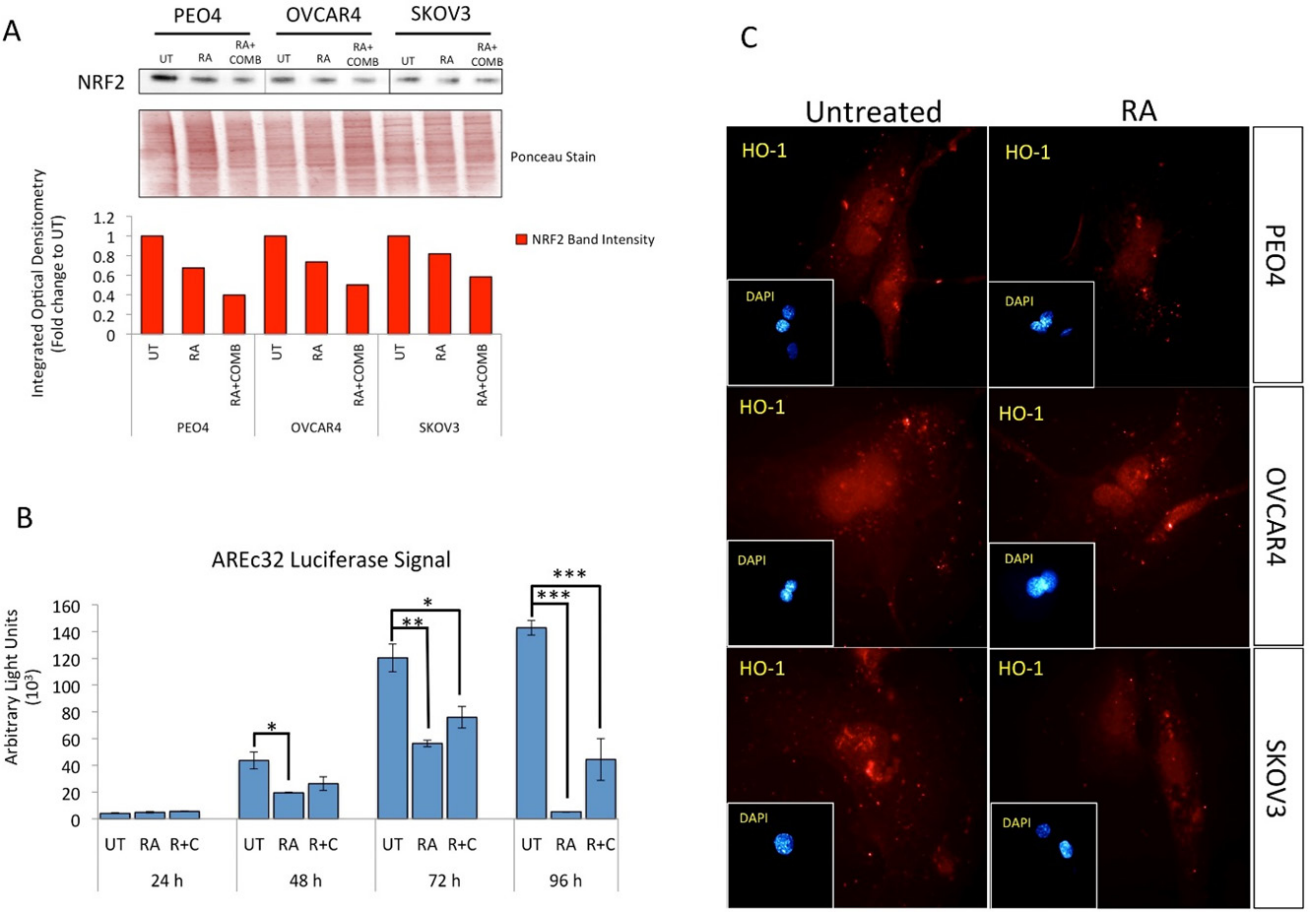
Treatment with Retinoic acid (RA) causes inhibition of NRF2 dependent antioxidant response pathway and generates ROS **(A)** Western analysis showing repression of NRF2 levels following RA treatment in PEO4, OVCAR4 and SKOV3 cell lines. Exponentially growing cells were either left untreated, treated with 2.5μM RA or a combination of 2.5μM RA together with 20μg/mL of Pertuzumab and Trastuzumab for 96 h before being harvested to prepare protein lysates and processed as described in Materials and Methods. Ponceau stain of the same blot was used as loading control. Red bars indicate NRF2 levels following quantification of immunoblot signal intensities obtained in (A) and normalized to the value of UT and expressed as fold change. The signal intensities of bands were quantified through integrated optical densitometry measurement. **(B)** RA treatment causes inhibition of NRF2 dependent transcription. Exponentially growing AREc32 cell line stably expressing 8x*cis*-antioxidant response elements driving the expression of luciferase gene in an NRF2 dependent manner were either left untreated (UT), treated with RA alone, or with RA and combination of Pertuzumab and Trastuzumab for different time points as indicated. Following this, cell lysates were prepared and assayed for Luciferase activity (BrightGlo Luciferase system, Promega). Data are the mean values ± S.D of quadruplicates, normalized to untreated (UT) and expressed as fold change with statistical significance determined by ONE WAY ANOVA followed by Tukey's post hoc test according to the scale * P <0.05, **P <0.01, ***P <0.001. **(C)** RA treatment causes repression of HO-1 levels. Immunofluorescent labelling of endogenous HO-1 in cells previously grown on poly-L lysine coated coverslips and exposed to RA treatment as in (A). For immunolabelling, primary HO-1 antibody followed by Alexa Fluor® 568 conjugated secondary antibody (red fluorescence) was used. Nuclear reference was provided by co-staining with 4′,6-Diamidino-2-Phenylindole, Dihydrochloride (DAPI). Images were captured with Leica DMiRe2 electronic microscope using integrated features of ANDOR iQ core software (ANDOR Technologies Ltd). Scale bar indicates 10μm. These are representative images taken in different field of views with relevant fluorescence channels and 100x objective.

**Figure 3:**
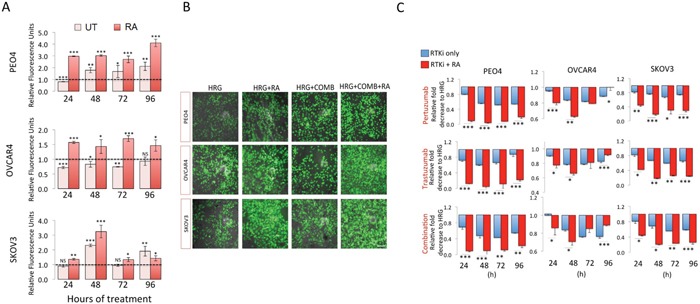
Inhibition of NRF2 pathway by Retinoic acid (RA) sensitizes ovarian cancer cells to immunotherapeutic agents targeting HER2 by increased ROS and enhanced growth inhibition **(A)** RA treatment causes increase in ROS levels. Exponentially growing cells were seeded in triplicates in opaque flat bottom black walled 96-well plates for 24 h. Following this, cells were either left untreated (UT) or treated with 1nM Heregulin and exposed to 2.5μM RA for different time points as indicated. Following incubations, cells were loaded with DCFDA fluorescent stain for 45 min and assayed for ROS by measuring fluorescence as described in Materials and Methods. Data is shown as fold change of RA treated cells to UT or RA and HRG treated cells to HRG treated cells as indicated (broken line). **(B)** Green fluorescent signal of DCFDA stain indicating ROS accumulation in ovarian cancer cells. Exponentially growing cells were either left untreated in media containing 1nM Heregulin (HRG) in the presence 5% charcoal stripped FBS, or treated with same media containing 20μg/mL of HER2 inhibitors, Pertuzumab and Trastuzumab either alone or in combination (RTKi only group) or in the continuous presence of 2.5μM RA (RTKi + RA group). Following treatments, cells were washed with PBS and imaged using Leica DMiRe2 electronic microscope and 485/535nm filter set. Merging with bright field phase contrast images captured in same field of view were performed by using integrated features of ANDOR iQ core software (ANDOR Technologies Ltd). Scale bar indicates 50μm. These are representative images with a 40x objective. **(C)** Growth inhibition measured using the MTT assay as described in Materials and Methods. Values are mean values ± S.D of triplicates and expressed as fold decrease to HRG. Statistical significance was determined between pairs of RTKi only and RTKi + RA groups by independent t test and significance expressed according to the scale * P <0.05, **P <0.01, ***P <0.001.

Retinoic acid (RA) has previously been shown to inhibit the antioxidant response (AR) pathway in an NRF2 dependent manner [53]. In order to extend the observations reported in the previous section, we wanted to study the consequences of NRF2 inhibition on survival following exposure to the HER2 targeting drugs. Firstly, we did a series of experiments in the ovarian cancer cell line models in order to validate and confirm the inhibitory action of RA on the NRF2 dependent AR pathway. Exposure to RA alone caused a decrease in total NRF2 levels (Figure 2A). Interestingly the levels of NRF2 in these cell lines were further decreased following co-treatment with combined immunotherapy (Trastuzumab & Pertuzumab). This drug induced reduction in NRF2 levels suggested that immunotherapy is also targeting NRF2. Next, using the luciferase ARE reporter AREc32 cell line, we demonstrated that RA treatment significantly inhibited transcriptional activity of NRF2 at all the time points tested (Figure 2B). RA treatment of AREc32 reporter cell line also elevated ROS levels (Supplementary Figure S1). Furthermore, RA could not further enhance the inhibitory action of combination of immunotherapeutic agents on AR pathway. We also examined the effect of RA treatment at single cell level on NRF2 substrate, HO-1, and could demonstrate a decrease in its abundance (Figure 2C). These findings suggested that while RA inhibits NRF2 dependent AR pathway, such treatment might also elevate cellular ROS levels in the ovarian cancer cell lines. Indeed we found that treatment with RA significantly induced ROS in the three cell lines tested (Figure 3A and 3B).

We next asked whether RA dependent inhibition of NRF2 AR pathway would sensitize ovarian cancer cells to targeted immunotherapeutic agents and if such treatment could achieve sensitization in the otherwise drug resistant OVCAR4 cell line. To do this, we repeated drug treatments either alone or in combination for 24-96 h, but this time with co-treatment of RA (Figure 3C). We found significantly enhanced cytotoxicity of targeted therapies following NRF2 inhibition in all three cell lines, in all treatments and at most time points tested. PEO4 cell line was most sensitized to such treatments with all groups showing significant increase in cell death. OVCAR4, which was more resistant, was also sensitized to targeted therapies following RA treatment. We also determined whether treatment with RA in the absence of any other drugs alone could curb cancer growth, and thus analyzed growth rates for 4 days and found growth inhibition (Supplementary Figure S2). These findings illustrated the important role of NRF2 in influencing outcomes to targeted therapies involving HER2 receptor inhibition.

These results indicate that effectiveness of anticancer therapy involving targeted immunotherapeutic agents could be enhanced by parallel inhibition of the NRF2 dependent antioxidant response pathway. As such, this represents a novel drug target in the context of HER2 inhibition and could explain the enhanced effectiveness of combination of Pertuzumab and Trastuzumab, a treatment that reduced NRF2 levels, as opposed to single agents. This could further serve to explain why HER2 immunotherapeutics in certain contexts show poor efficacy.

### Action of targeted immunotherapeutic agents involves repression of NRF2-dependent transcription and depletion of total Glutathione

**Figure 4:**
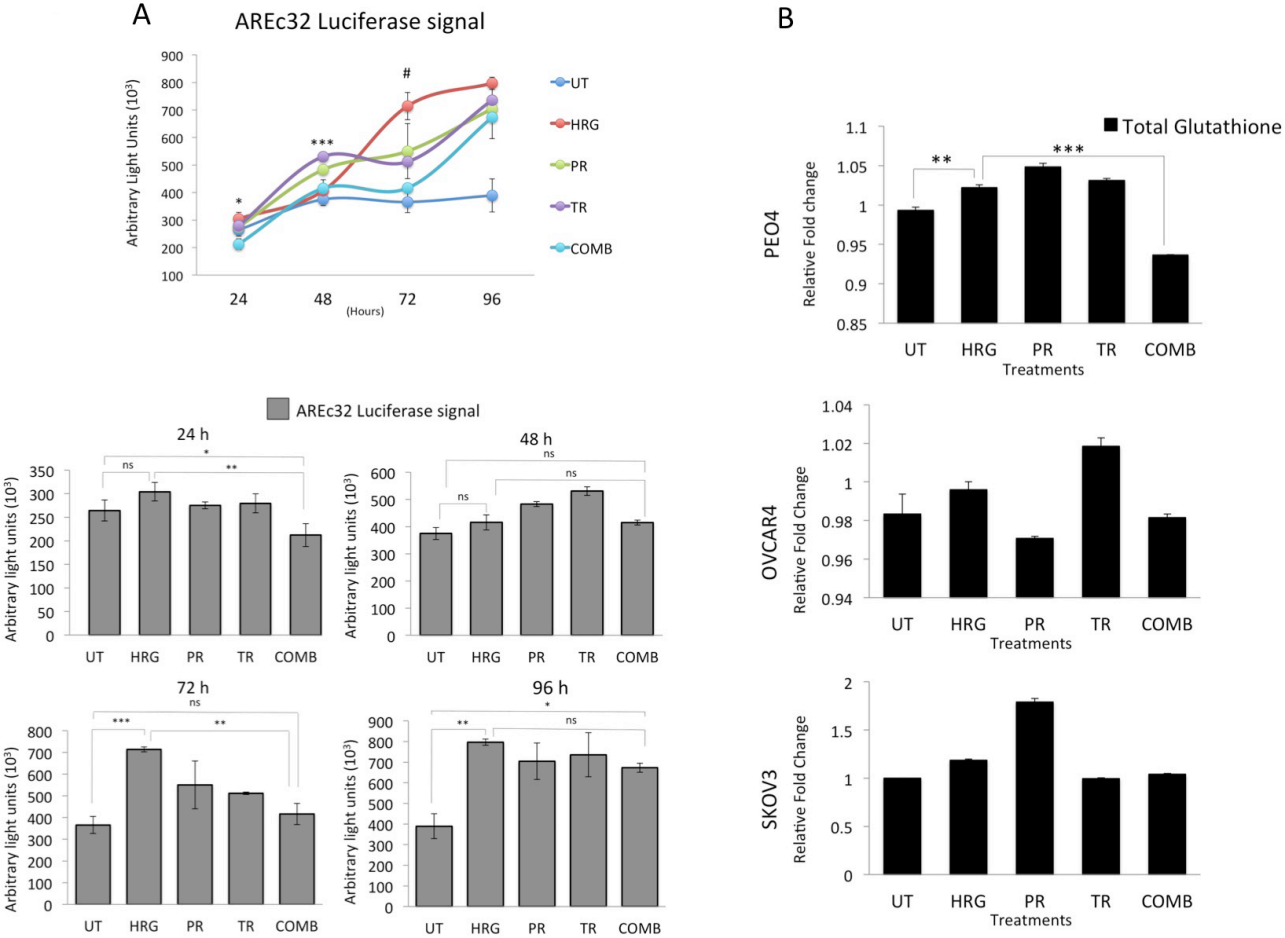
Treatment with Pertuzumab and Trastuzumab causes repression of NRF2 dependent transcription and depletion of total Glutathione levels **(A)** Combination of Pertuzumab and Trastuzumab cause inhibition of NRF2 dependent transcription. Exponentially growing AREc32 cell line stably expressing *cis*-regulatory antioxidant response elements driving the expression of luciferase gene in an NRF2 dependent manner were treated with 1nM HRG alone or with co-treatment of 20μg/mL Pertuzumab and Trastuzumab either individually or in combination for different time points as indicated. Following this, cell lysates were prepared and assayed for Luciferase activity as described in Materials and Methods. Data shown are mean values ± S.D of quadruplicates, normalized to untreated (UT) and expressed as fold change with statistical significance determined by ONE WAY ANOVA followed by Tukey's post hoc test. In upper panel, asterisks indicate significant difference in inhibition between monotherapy and combination while the # notation indicates the significant difference of inhibition between HRG and all the treatment types. In lower panel, asterisks indicate significant differences between individual groups as indicated and according to the scale * P <0.05, **P <0.01, ***P <0.001. **(B)** Combination of targeted immunotherapeutic agents causes glutathione depletion. Exponentially growing cells were seeded in 60mm tissue culture plates for 24 h and either left untreated (UT) or treated with media containing 1nM Heregulin alone (HRG) or with co-treatment of 20μg/mL Pertuzumab (PR), Trastuzumab (TR) or their combination (COMB) for 96 h before being harvested to prepare protein lysates and processed for glutathione assay. Data were normalized to the total protein content from same plates determined by Bradford assay and are mean values ± S.D of triplicates and expressed as fold change to the UT. Statistical significance was determined by ONE WAY ANOVA followed by Tukey's post hoc test according to the scale * P <0.05, **P <0.01, ***P <0.001.

Having observed additional decreased levels of NRF2 in these cell lines following combined immunotherapy (Trastuzumab & Pertuzumab) with RA treatments, we next asked whether targeted immunotherapy would also inhibit NRF2-dependent transcription. To address this, we used the AREc32 cell line, stably expressing 8 copies of NRF2 dependent cis-regulatory antioxidant response elements, as a luciferase reporter. We found that while HRG stimulation alone induced the antioxidant response pathway, co-treatment with combination of Pertuzumab and Trastuzumab not only disrupted such induction, but further suppressed it significantly at 24 and 72 h of treatment (Figure 4A). Treatments with single agents alone also inhibited NRF2 function, although to a lesser extent than their combination.

We next asked whether NRF2 repression would also lead to depletion of total cellular glutathione. A 96 h treatment with HRG induced total cellular glutathione, while combination of Pertuzumab and Trastuzumab significantly reduced this level in PEO4 cells. The same was seen for OVCAR4 and SKOV3 cells, albeit not significantly (Figure 4B). These studies demonstrate that HER2 targeting monoclonal antibodies repress the NRF2 dependent antioxidant pathway which may well contribute to the unique enhanced cytotoxicity in the combination of Pertuzumab and Trastuzumab.

### Ovarian cancer cells exhibit different degrees of cytotoxicity to HER2 targeting immunotherapeutic agents Pertuzumab and Trastuzumab

**Figure 5:**
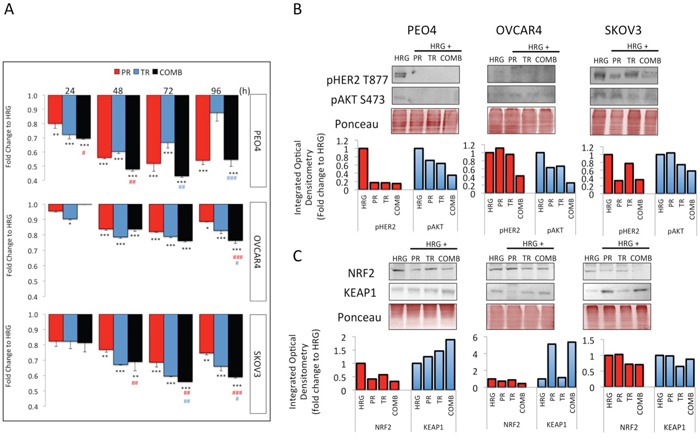
Treatment with Trastuzumab and/or Pertuzumab causes cytotoxicity and modulates expression of pHER2, pAKT, NRF2 and KEAP1 in ovarian cancer cell lines **(A)** Effect of antibodies on cell number. Cells were treated with Pertuzumab (PR), Trastuzumab (TR) or combination (COMB) of antibodies in the presence of 1nM Heregulin. Antibodies were used at 20μg/mL. After treatment, cell number was assessed by use of the MTT assay as described in Materials and Methods. Values are means with ± S.D of triplicates and expressed as fold decrease to HRG alone. Statistical significance was determined between untreated and treated groups by ONE WAY ANOVA followed by Tukey's post hoc test. Asterisks indicate significance of difference between HRG and individual treatment group whereas “#” indicates significance of difference between combination and Pertuzumab (red sign) or combination and Trastuzumab (blue sign) according to the scale * P <0.05, **P <0.01, ***P <0.001. **(B)** Western blot analysis showing levels of phospho HER2 T877 and phospho AKT S487 and **(C)** NRF2 and KEAP1 in response to treatments with 20μg/mL of Pertuzumab (Pr), Trastuzumab (Tr) and their combination (Comb) in PEO4, OVCAR4 and SKOV3 cells. Exponentially growing cells were treated with media containing 1nM Heregulin alone, or with co-treatment of Pertuzumab, Trastuzumab or their combination for 96h before being analyzed as described in Materials and Methods. The bar charts show phospho HER2 or NRF2 (red bars) and phospho AKT levels or KEAP1 (blue bars) following quantification of the immunoblot signal intensities obtained and normalized to HRG treatment group and expressed as fold change. The signal intensities of bands were quantified through integrated optical densitometry measurement using Gelpro software (Version 3.1, Media Cybernetics).

We next determined the degree of sensitivity of ovarian cancer cells derived from different origins to the HER2 targeting monoclonal antibodies, Pertuzumab and Trastuzumab, either used individually or in combination (Figure 5A). To do this, we again used the low HER2 expressing ovarian cancer cell line, OVCAR4, the moderately HER2 expressing PEO4 and the HER2 overexpressing cell line, SKOV3 [34]. Moreover, the expression of the HER2 key heterodimerization partner, HER3, is variable in these ovarian cell lines with PEO4 expressing the highest, OVCAR4 moderate and SKOV3, the lowest [34]. We found that not only did the cells exhibit variable and statistically significant and distinct susceptibilities to the monoclonal antibodies, but that they also exhibited time dependent variation in sensitivities (Figure 5A). For example, within single treatments, while Pertuzumab was more cytotoxic (than Trastuzumab) to PEO4 cells, Trastuzumab achieved greater cytotoxicity in OVCAR4 and SKOV3 cell lines. Secondly, with each treatment modality, growth inhibition was maximal following 72 h of treatment. Thirdly, the combination treatment, having both Pertuzumab and Trastuzumab, was generally more cytotoxic than single agents in all cell lines. Finally, overall, PEO4 was found to be more sensitive to combination of the drugs, SKOV3 showed moderate sensitivity while OVCAR4 was least sensitive (Figure 5A).

This analysis revealed the differential responses of the tested ovarian cancer cell lines to the same HER2 inhibitors indicating the variable nature of outcomes to targeted therapy involving HER2 inhibitors. However, since most of the time points tested revealed the combination of the two inhibitors produced the highest growth inhibition, this initial cytotoxicity data supports the use of the combination treatment in ovarian cancer [31, 33].

### Pertuzumab and Trastuzumab produce different levels of RTK signaling inhibition in ovarian cancer cell lines

Upon observing varying degrees of growth inhibition to Pertuzumab and Trastuzumab in the three cell lines used, we next sought to determine whether these also achieve different degrees of RTK signaling inhibition. Specifically, we determined the levels of phosphorylated forms of HER2 Thr 877 (pHER2) and AKT Ser 473 (pAKT) following 96 h treatment with the HER2 targeting agents (Figure 5B).

At 96 h, there was more marked repression of pHER2 and pAKT in the PEO4 cell line than either OVCAR4 or SKOV3 cells, consistent with the observation of increased sensitivity of PEO4 to these inhibitors. Furthermore, treatment with a combination of the two agents usually achieved the highest inhibition of RTK signaling, again consistent with the highest growth inhibition seen in combination treatment in Figure 5A. Finally and again consistent with Figure 5A, OVCAR4 was found to have least inhibition of the phosphorylated substrates at all time points tested.

NRF2 is a master regulator of the antioxidant response (AR) pathway [7], while KEAP1 is its main regulator by functioning as an adaptor for ubiquitin ligase complex and promoting NRF2 degradation [54]. In the context of targeted immunotherapy, recent reports have suggested a role for NRF2 in determining overall treatment response [37, 55, 56]. More specifically, several studies have either suggested direct cross talk of NRF2 and RTK signaling [36], or modulation of NRF2 by the RTK substrates PI3K [57–59] and MAPK [42, 43]. Furthermore, both RTK mediated growth promoting pathways and NRF2 dependent antioxidant responses have been documented to lead to drug resistance of cancer cells [60–64]. In light of these emerging roles of NRF2 in determining overall treatment responses, we next extended our investigation to study regulation of NRF2 and its degrader, KEAP1, in response to targeted therapies.

Treatment with HER2 inhibitors either alone or in combination on the contrary reduced NRF2 levels equally in our cell lines, consistent with previous studies [36]. The combination treatment caused a distinct repression in NRF2 levels in all three cell lines as compared to HRG treatment alone, whereas at the same time point, there was induction of KEAP1 in PEO4 and SKOV3 cell lines (Figure 5C). Both NRF2 repression and KEAP1 induction were greater in PEO4 and SKOV3 cell lines than in OVCAR4, consistent with the cytotoxicity profiling (Figure 5A). NRF2 is implicated in numerous contexts in causing drug resistance [62–65] and the observed repression of NRF2 following combination treatment and greater repression in more sensitive cell lines is again consistent with a role for NRF2 in determining sensitivity to these targeted agents.

To characterize these observations further in an *in vivo* context, we performed extensive bioinformatic analysis and data mining from *in vivo* SKOV3 xenograft model previously exposed to same HER2 target immunotherapies. We especially sought to determine the precise mechanism of combination-induced repression of NRF2 and its antioxidant function.

### Genetic reprogramming and NRF2 signaling following *in vivo* anti-HER2 immunotherapy

We recently reported on some genetic cellular reprogramming events following HER2-targeted immunotherapy with Trastuzumab or Pertuzumab and their combination in an *in vivo* xenograft model of ovarian cancer [25, 32]. We re-examined the expression profile and microarray data previously reported [32, 48]. However, this time we digressed from global analysis of gene expression and focused on the NRF2 network using a knowledge-based approach that is largely informed by information reported in [50–54]. We analyzed gene expression data as before [28, 32] on the response of SKOV3 xenograft tumors to Trastuzumab, Pertuzumab, and their combination treatments in the context of dynamic changes in the NRF2 network and signaling pathway.

**Figure 6:**
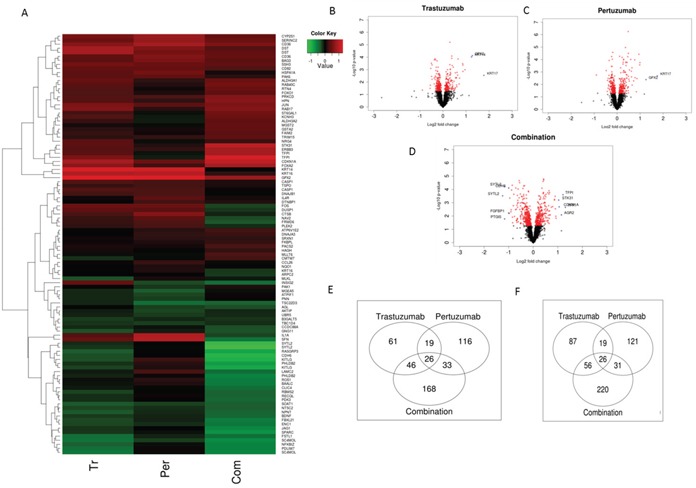
NRF2 network dependent molecular responses to Trastuzumab and Pertuzumab alone or in combination **(A)** Heatmap showing significant (p=0.001) differential expression of genes within the NRF2 network relative to control treatment of SKOV3 ovarian xenograft tumors (SAM FDR=10%) as in References [15, 48]. Red represents increased expression and green decreased expression relative to the median of the controls. **(B-D)** Volcano-plots showing gene expression changes within the NRF2 network following Trastuzumab (B), Pertuzumab (C), and their combination (D) treatment of the SKOV3 xenograft tumors. The genes with significant (*p* ≤ 0.05) and non-significant fold changes are colored red and black, respectively, while some genes with a high fold change are marked by blue crosses. **(E-F)** Venn diagram showing distinct and overlap of significantly up-regulated (E) and down-regulated (F) transcripts of genes within the NRF2 network relative to control treatment of SKOV3 xenograft tumors with Trastuzumab and Pertuzumab alone or in combination.

A total of 3599 transcripts of 2250 genes linked to the NRF2 network and function were identified in the array with 14, 15 and 24% either upregulated or down regulated significantly (p≤0.05) following Trastuzumab, Pertuzumab and combination of the HER2-targeted immunotherapies (Figure 6A; Supplementary Table S1 and S2). The volcano plots illustrated in Figure 6B–6D indicate transcripts of genes within the NRF2 network. Strikingly, we identified significant changes in expression within the genes of the NRF2 network following immunotherapies relative to control values. Many of the differentially expressed genes in the NRF2 network due to combination treatment were already modulated by either Trastuzumab and/or Pertuzumab monotherapy. The Pertuzumab and combination treatments produced greater effects in gene expression changes in ascending order of magnitude relative to Trastuzumab, both in terms of significant (p≤0.05) down- and up-regulation of genes (Figure 6E and 6F; Supplementary Table S1 and S2). Specifically some of these transcripts were significantly down regulated (p≤0.05) following immunotherapy of which 87, 121, and 220 were due to Trastuzumab, Pertuzumab and combination of respectively. Furthermore, a total of 19, 31, and 56 genes and transcripts were commonly and significantly down regulated (p≤0.05) by Trastuzumab and Pertuzumab, by Pertuzumab and combination, and by Trastuzumab and combination, respectively. The significant down regulation (p≤0.05) of 26 genes was common to both single and combination therapies. Irrespective of either monotherapy or combination therapy, within the NRF2 network, the total up- and down-regulated genes, especially of the significant ones (p≤0.05) by each therapy were closely balanced and symmetrical (Figure 6B–6D, also see Supplementary Table S2). However, significantly greater volcanicity was observed with combination immunotherapy. Overall a total of 309, 354, and 540 genes were significantly affected (p≤0.05) by Trastuzumab, Pertuzumab, and their combination, which accordingly represent 1:1.2:1.8 fold differences in the number of affected genes within the NRF2 network following these immunotherapies, respectively. We could visualize these significant changes in the NRF2 network using heatmaps, however, due to the large number of the genes affected, we chose to present only the filtered heatmap showing genes that remained significant even at p=0.001 (Figure 6A). The higher number of modulated genes recorded with the combination therapy relative to the monotherapies is indicative of a more complex gene expression changes and cellular reprogramming events, which led to the observed greater cellular ROS production and compromise of cellular NRF2 levels and functions.

More specifically, genes within the NRF2 network that were markedly expressed and down regulated following combination immunotherapy were detoxification and metabolism related *AKR1B1, AKR1C1, ATP13A3, ATP1B1, ATP2A1, HMOX1, NQO1, FRMD6, GAPDH, GCLM, GCSH, GSTM4, H6PD, IDH1, PRDX1, SOAT1, SOD2, UGDH*; DNA damage and repair related *MLH3, MSH6, MYH4*; cell signaling, proliferation, inflammation and immunity, and angiogenesis, related *AKT3, AKTIP, CDH6, CXXC5, CXCL2, GSK3A, GSK3B, HACE1, ID1, KIT, KITLG, OSMR, PIK3AP1, PIK3IP1, PIK3R2, PIK3R5, RPS6KB1; EPHA2, FGF12, FGFBP1, FOS, FOSL2, FSTL1, IGF2BP3, JUNB, MAPK1, NGF, PDGFA, PDGFC, PDGFD, TGFA, TNFRSF1A, TAX1BP1, TUBB3, VEGFA*, cell cycle regulation, cell survival and death related *BAD, BARD1, BCL6, BCL2L15, BNIP1, CDK7, CDKN3, NAV2*; as well as transcription and epigenetics control related *AHR, ARNTL, BACH1, BACH2, BRCA1, BRCA2, ETS1, NFE2L2, NFKBIZ, RORA, RUNX2*; *BLM, HAT1, HMGB1, MED4, MET, METRN, METTL1, PRRX1, TES* (Supplementary Table S1 and S2; Figure 6A). Likewise some genes within the NRF2 network that were markedly expressed and up regulated following combination immunotherapy were detoxification and metabolism related *ABCB9, ABCC1, ABCC10, ACOX2, GPX2, GPX4, GSR, GSS, GSTA2, GSTM1, GSTM2, GSTM3, TALDO1, UGT1A6*; cell signaling, proliferation, inflammation and immunity, and angiogenesis, related *AKT1, CEBPA, DUSP5, DUSP5P, DUSP6, ERBB2, ERBB2IP, ERBB3, FOXA2, FOXJ2, FOXJ2, FOXO1, FOXO3, JUN, NFE2L1, PDGFB, RPS6KA2, RPS6KB2*; cell cycle regulation, cell survival and death related *CDKN1A, CDKN1B, CDKN2A, CDKN2B, CASP1, FRMD6, VAV3*; as well as stability, transcription and epigenetics control related BACH1, BACH2, BTRC, KEAP1, MAFF, MAFG, MAFK, NOTCH1, NOTCH3, PRRX2, NCOA6, NCOR1, RARA, RCOR3, RORA, RXRA, RXRB, RXRG, SYK, XBP1, HDAC1, HDAC3, HDAC5 (Supplementary Table S1 and S2; Figure 6A). Some similarly related gene expression changes were seen with single immunotherapy, Trastuzumab or Pertuzumab, but sometimes to a different magnitude and different direction.

**Figure 7:**
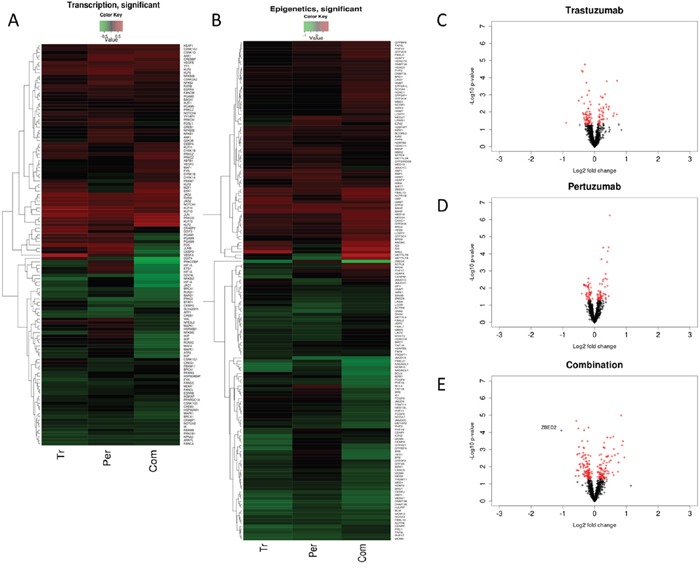
Common and differential expression changes in transcription and epigenetics associated genes in responses to Trastuzumab and Pertuzumab alone or in combination of **(A)** Heatmap showing differential changes **(B)** Heatmap showing significant (p≤0.05) differential changes in the expression of the genes relative to control treatment of SKOV3 ovarian xenograft tumors (SAM FDR=10%) as in References [15, 48]. Red represents increased expression and green decreased expression relative to the median of the controls. **(C, D)** Volcano-plots showing gene expression changes in transcription and epigenetics associated genes following trastuzumab (C), pertuzumab (D), and their combination **(E)** treatment of the SKOV3 xenograft tumors. The genes with significant (*p* ≤ 0.05) and non-significant fold changes are colored red and black, respectively, and some genes with a high fold change are marked by blue crosses.

**Figure 8:**
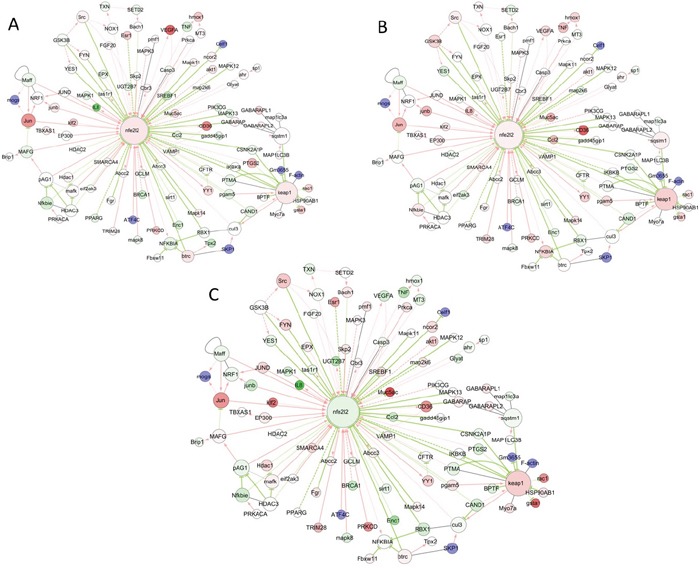
Visualization of common and differential gene expression changes within the NRF2 network and signaling pathways Network of gene expression profiles in response to either Trastuzumab **(A)** or Pertuzumab **(B)** alone, or following their combination treatment **(C)** in SKOV3 xenograft tumors from References [15, 48], and as depicted in the NRF2-related interactome and regulome model of Papp D et al 2012 [50]. The color of the node depicts the ratio of the expression values with drug compared to the expression values in control samples.

**Figure 9:**
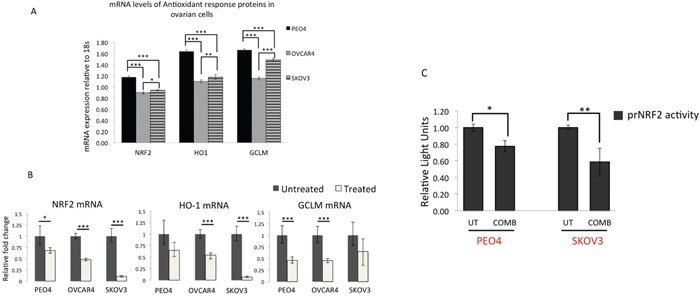
qRT PCR analysis of NRF2 and its substrates reveal downregulation of *NRF2, HO-1* and *GCLM* mRNA expression following treatment with targeted immunotherapeutic agents **(A)** Ovarian cancer cell lines exhibit different basal levels of NRF2 and its substrate expression. Exponentially growing cells were treated with 1nM Heregulin for 96 h. Following this, cells were harvested for total RNA for subsequent reverse transcription to generate cDNA and used to perform quantitative Real Time PCR (qRT PCR) as described in Materials and Methods. **(B)** Treatment with combination of Pertuzumab and Trastuzumab causes downregulation of NRF2, HO-1 and GCLM mRNA. Cells were exposed to combination of 20μg/mL of Pertuzumab and Trastuzumab for 96 h before being harvested for RNA and subjected to qRT PCR as in (A). **(C)** Luciferase reporter assay for NRF2 transcription demonstrates repression of NRF2 transcription following exposure to HER2 targeting immunotherapeutic agents. Exponentially growing PEO4 and SKOV3 cells were transfected with either empty PGL3 basic vector or 1μg PGL3 basic vector with cloned 1.5kb fragment of NRF2 promoter (prNRF2) driving the expression of luciferase gene. Co-transfection with 0.2μg pRL-CMV plasmid was performed as an internal transfection control as described in the Materials and Methods. At 24 h post-transfection, cells were either left untreated in media containing 1nM HRG (UT) or treated with combination of 20μg/mL of Pertuzumab and Trastuzumab (COMB) for 96 h. Following treatments, cell lysates were prepared from the transfected cells, transferred to opaque flat bottom white 96-well plates and their luciferase activity recorded using Dual luciferase reporter assay (Promega) in multiplate luminometer (MODULUS^™^, Promega). For (A), (B) and (C), data are means with ± S.D of triplicates and expressed as fold change with statistical significance determined by either ONE WAY ANOVA or independent *t* test as appropriate according to the scale * P <0.05, **P <0.01, ***P <0.001.

**Figure 10:**
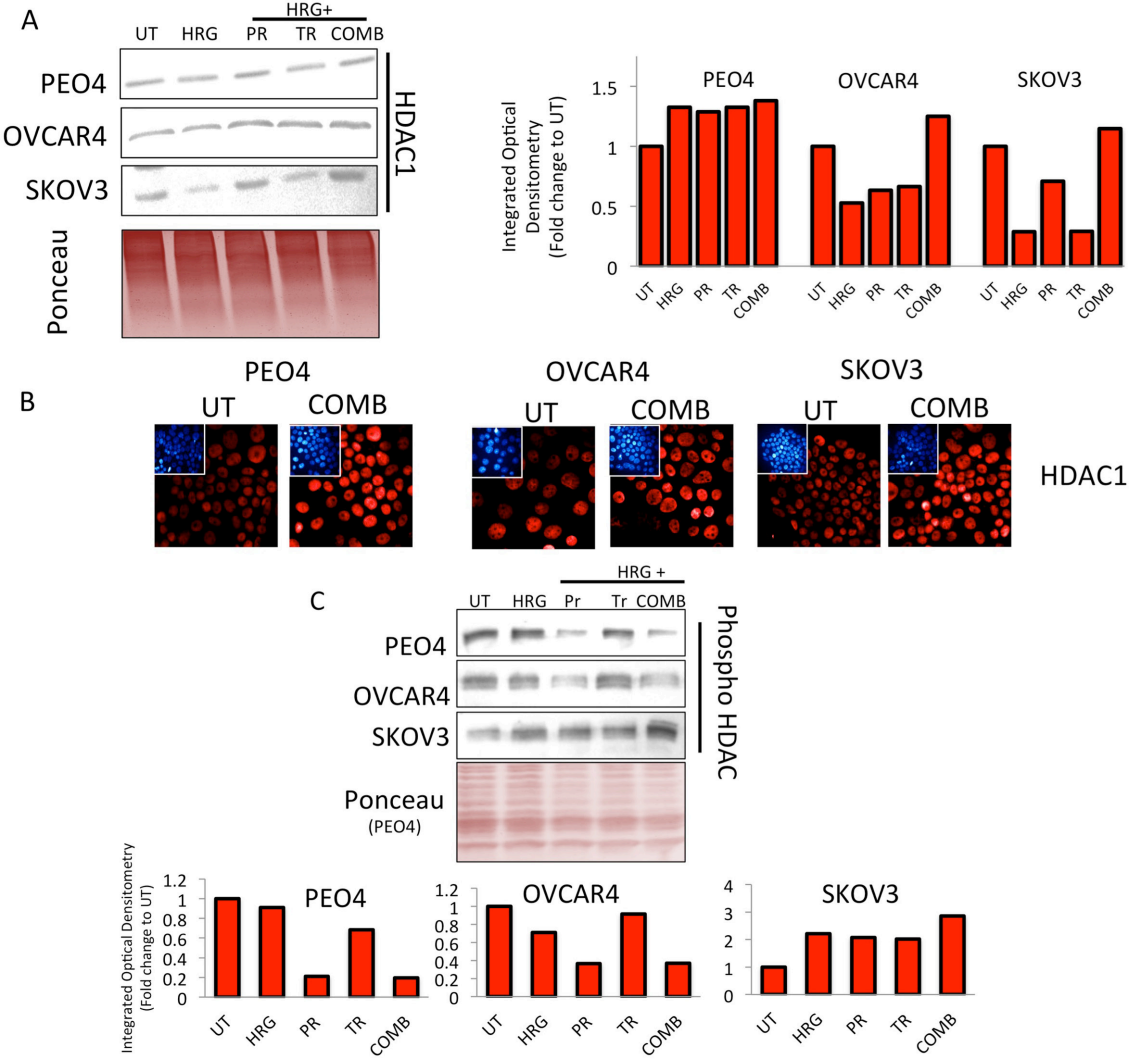
Treatment with HER2 targeting immunotherapeutic agents causes induction of HDAC1 expression and repression in phospho-HDAC levels **(A)** Immunoblotting analysis showing induction of total HDAC 1 levels following treatment with combination of Pertuzumab and Trastuzumab in PEO4, OVCAR4 and SKOV3 cell lines. Exponentially growing cells were either left untreated (UT) or treated with media containing 1nM Heregulin alone (HRG), or with co-treatment of 20μg/mL of Pertuzumab (PR), Trastuzumab (TR) or their combination (COMB) for 96 h before being harvested for immunoblotting using an anti-HDAC1 antibody (Table 1). Ponceau stain of the same blot was used as loading control Red bars indicate HDAC1 levels following quantification of the immunoblot signal intensities obtained and normalized to the value of UT and expressed as fold change. The signal intensities of bands were quantified as described in Materials and Methods, **(B)** Treatment with HER2 inhibitors causes induction of nuclear HDAC1 levels. Immunofluorescent labelling of endogenous HDAC1 in cells previously grown on poly-L lysine coated coverslips and exposed to treatments as in (A). For immunolabelling, Alexa Fluor® 568 conjugated secondary antibody (red fluorescence) was used. Nuclear reference was provided by co-staining with 4′,6-Diamidino-2-Phenylindole, Dihydrochloride (DAPI). Images were captured with Leica DMiRe2 electronic microscope using integrated features of ANDOR iQ core software (ANDOR Technologies Ltd). Scale bar indicates 10μm. These are representative images taken in different field of views with relevant fluorescence channels and 100x objective. **(C)** Immunoblotting analysis of phospho-HDAC levels following treatment with combination of Pertuzumab and Trastuzumab in PEO4, OVCAR4 and SKOV3 cell lines. Exponentially growing cells were either left untreated (UT) or treated with media containing 1nM Heregulin alone (HRG), or with co-treatment of 20μg/mL of Pertuzumab (PR), Trastuzumab (TR) or their combination (COMB) for 96 h before being harvested to prepare protein lysates and processed for immunoblotting using an anti-phospho HDAC antibody (Table 1). Ponceau stain of the same blot was used as loading control (not shown). Red bars indicate phospho-HDAC levels following quantification of the immunoblot signal intensities obtained in (A) and normalized to the value of UT and expressed as fold change. The signal intensities of bands were quantified through integrated optical densitometry measurement as described in Materials and Methods.

**Figure 11:**
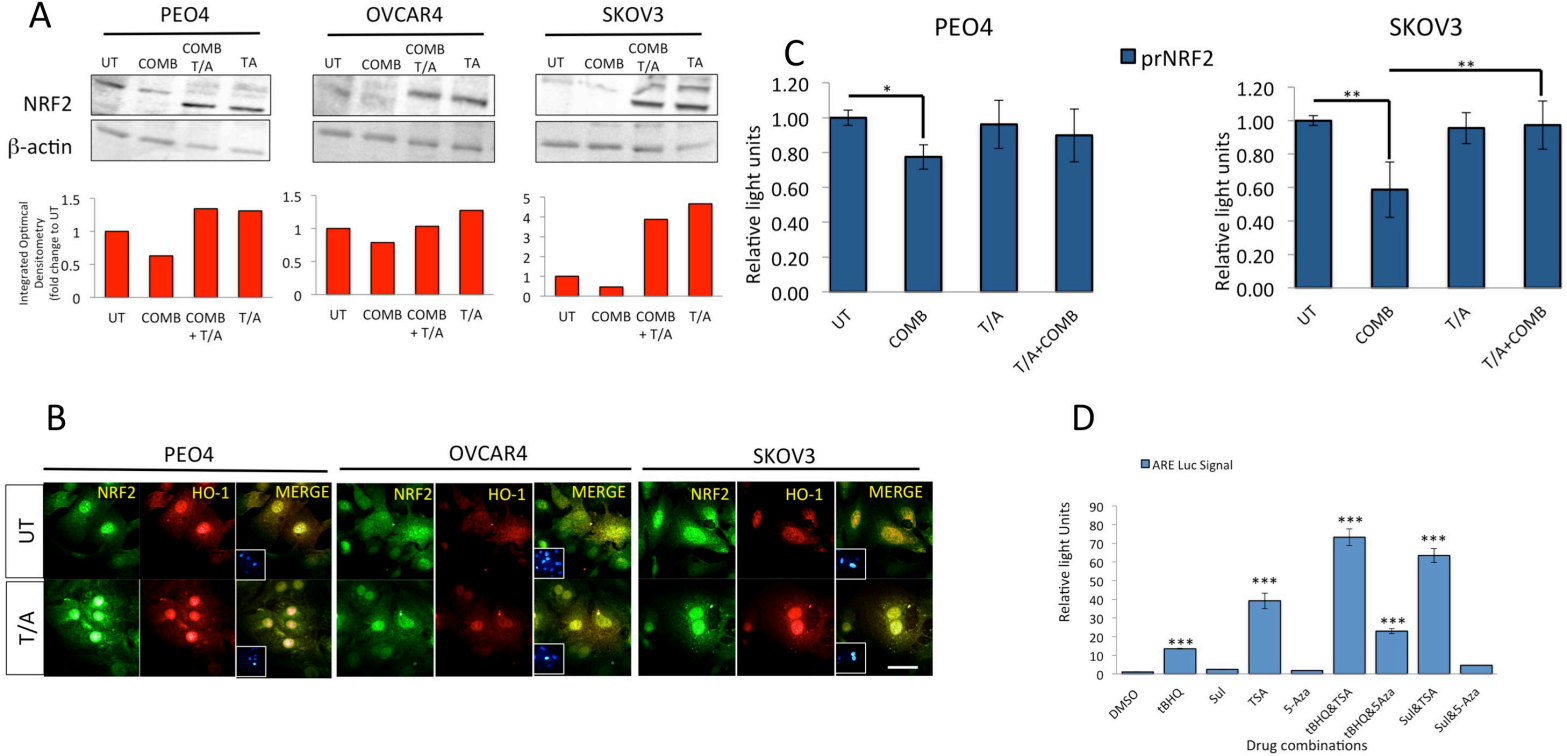
Inhibition of HDAC and DNA Methyl Transferase induce NRF2 protein levels, causes nuclear localization of NRF2 and HO-1, disrupts both targeted immunotherapy dependent NRF2 protein and transcriptional repression, and activates NRF2 dependent antioxidant transcriptional response program **(A)** Immunoblotting analysis showing induction of total NRF2 levels following treatment with combination of Trichostatin and 5-azacytidine both in the absence and presence of HER2 inhibitors. Exponentially growing cells were either left untreated in media containing 1nM Heregulin alone (UT) or treated with either a combination of 20μg/mL Pertuzumab and Trastuzumab (COMB), with combination of 200nM Trichostatin A and 2μM 5-Azacytidine (T/A) or with all four drugs together for 96 h. Following incubation, cells were harvested to prepare protein lysates and processed for immunoblotting using relevant antibodies (Table 1). Blotting with β-actin was used as loading control. Red bars indicate NRF2 levels following quantification of the immunoblot signal intensities obtained in (A) and normalized to the value of UT and expressed as fold change. The signal intensities of bands were quantified through integrated optical densitometry measurement using Gelpro software (Version 3.1, Media Cybernetics). **(B)** Treatment with T/A causes nuclear induction of NRF2 and HO-1 levels. Immunofluorescent labelling of endogenous NRF2 (upper panels) and HO-1 (lower panels) in cells previously grown on poly-L lysine coated coverslips and exposed to T/A treatments as in (A). For immunolabelling, Alexa Fluor® 488 conjugated secondary antibody was used for NRF2 staining (green fluorescence), while Alexa Fluor® 568 conjugated secondary antibody was used for HO-1 staining (red fluorescence). Nuclear reference was provided by co-staining with 4′,6-Diamidino-2-Phenylindole, Dihydrochloride (DAPI). Images were captured with Leica DMiRe2 electronic microscope, while merging, co-localization and further analysis were performed by using integrated features of ANDOR iQ core software (ANDOR Technologies Ltd). Scale bar indicates 10μm. These are representative images taken in different field of views with relevant fluorescence channels and 100x objective. **(C)** Inhibition of HDAC and DNA Methyl Transferase disrupt the transcriptional inhibition of NRF2 exerted by the HER2 targeting immunotherapeutic agents. Exponentially growing PEO1 and SKOV3 cells were seeded in triplicates in 24 well plates and transfected with either empty PGL3 basic vector or 1μg PGL3 basic vector with cloned 1.5kb fragment of NRF2 promoter (prNRF2) driving the expression of luciferase gene. Co-transfection with 0.2μg pRL-CMV plasmid was performed as an internal transfection control. At 24 h post-transfection, cells were either left untreated in media containing 1nM HRG (UT) or treated with combination of 20μg/mL of Pertuzumab and Trastuzumab (COMB), or combination of 200nM Trichostatin A and 2μM 5-Azacytidine (T/A) or combination of COMB and T/A as indicated for 96 h. Following treatments, cell lysates were prepared from the transfected cells, transferred to opaque flat bottom white 96-well plates and their luciferase activity recorded using Dual luciferase reporter assay (Promega) in multiplate luminometer (MODULUS^™^, Promega). **(D)** Inhibition of HDAC1 and DNA Methyl Transferases induce NRF2 dependent antioxidant transcriptional program. Exponentially growing AREc32 cell line stably expressing NRF2 dependent 8x*cis*- regulatory antioxidant response elements were seeded in triplicates in opaque 96 well plates and either mock treated (DMSO) or exposed individually to either 50μM *tert*-butylhydroquinone (tBHQ), 5μM Sulforophane (Sul), 5μM Trichostatin (TSA), 5μM 5-azacytidine (5-Aza), or a combination of tBHQ with either TSA or 5-Aza, or a combination of Sul with TSA or 5-Aza for 24 h. Following this, cell lysates were prepared and assayed for Luciferase activity (BrightGlo Luciferase system, Promega). For (C) and (D), Data are the means with ± S.D of triplicates and expressed as fold change with statistical significance determined by ONE WAY ANOVA followed by Tukey's post hoc test according to the scale * P <0.05, **P <0.01, ***P <0.001.

It is clear that Trastuzumab, Pertuzumab and combination have all produced significant induction of *KEAP1* expression. Whilst Trastuzumab and Pertuzumab caused significant induction of *NRF2* expression, their combination strikingly resulted in the profound and highly significant down regulation of *NRF2* expression. These *in vivo* observations strongly supported our *in vitro* data (Figure 5C) regarding the status of NRF2 and KEAP1 following immunotherapies. Further analyses and visualization of the *in vivo* gene expression data (Figure 7 and Figure 8) confirmed our observed perturbations in the NRF2 network and down regulation of some NRF2 target genes, especially genes associated with antioxidant responses and glutathione metabolism These give credence to our assertion of ROS production and compromise of NRF2 status and functions as the basis of action and effectiveness of the immunotherapies, especially with the combination of Trastuzumab and Pertuzumab. It is of interest to observe the up regulation of expression of *HDAC*s and certain nuclear co-repressor genes, as well as the down regulation of *HAT* expression and its related functional homologues or orthologues such as *DNA2, HES1, MED4, MET, METRN, METTL1*, which point to transcriptional packaging and control. Consequently, we hypothesized that transcriptional silencing and epigenetics may contribute to the distinct mechanisms of inhibition of NRF2 function by combination immunotherapy. In an attempt to test this hypothesis, we used KEGG and knowledge-based approach to examine the *in vivo* gene expression data with particular attention to transcription and epigenetics pathways. The data obtained (Figure 8) supported this hypothesis, which we later confirmed using our *in vitro* cell models (Figures 9–11). Overall there is agreement between the *in vitro* and the *in vivo* data.

### Combination treatment with Pertuzumab and Trastuzumab causes transcriptional repression of NRF2 leading to downregulation of expression of genes under its regulation

In order to verify the *in vivo* data of microarray and confirm transcriptional inhibition of NRF2 in our own cell line models, we adopted two approaches. Firstly, we performed quantitative RT-PCR (qRT-PCR) on cDNAs obtained from PEO4, OVCAR4 and SKOV3 previously exposed to combination treatment of Pertuzumab and Trastuzumab for 96 h. In this experiment, we firstly quantitatively determined basal expression levels of *NRF2* and its substrates *HO-1* and *GCLM* through qRT-PCR (Figure 9A). Consistent with previous reports, we found significantly higher basal levels of *NRF2* [31] as well as *HO-1* and *GCLM* levels in PEO4 than OVCAR4 and SKOV3, with OVCAR4 having the least expression levels. More importantly, following 96 h of treatment with combination of Pertuzumab and Trastuzumab, there was significant downregulation of *NRF2* expression in all the three cell lines tested. Furthermore, *HO-1* and *GCLM* expression were reduced following the same treatments (Figure 9B). This demonstrated and confirmed that targeted therapy cause transcriptional repression of *NRF2* leading to downregulation in expression of its transcriptional substrates and as such confirmed the *in vivo* microarray data.

In the second and related strategy, we cloned the 1.5kb promoter region of *NRF2* gene into a luciferase reporter vector to generate a luciferase based reporter assay for *NRF2* transcription (called prNRF2). This reporter was used to directly report any transcriptional perturbation of *NRF2*. We transfected prNRF2 into our ovarian cancer cell lines, repeated the immunotherapeutic combination treatment for 96 h and assayed cells for luciferase activity (Figure 9C). We found that indeed combination treatment significantly reduced luciferase signal, demonstrating transcriptional inhibition of *NRF2* expression in both PEO4 and SKOV3 cell lines. In this transient transfection strategy, by 96 h, we could not obtain any detectable expression of our vectors in OVCAR4 (data not shown). These experiments clearly demonstrated transcriptional inhibition of *NRF2* and explain the repression of NRF2 protein seen before (Figure 5C).

### HER2 targeting immunotherapeutic agents cause upregulation of HDAC1 and repression of phospho HDAC levels demonstrating HDAC activation

A noticeable feature in our *in vivo* microarray data analysis was the finding that several histone deacetylases (HDACs) levels were upregulated (Figure 7). Upregulation and activation of HDAC1 would lead to diminished acetylation, increased methylation and subsequent repression of gene expression [66, 67]. We next sought to determine whether upregulated HDAC1 could be reproduced in our cell lines following combination treatment *in vitro*, which could further serve to explain the transcriptional downregulation of *NRF2* expression during such treatments. Firstly, we immunoblotted for total HDAC1 in our three cell lines and saw induction of HDAC1 only in combination treatment, albeit to different levels in each cell line (Figure 10A). Furthermore, we fluorescently immunostained total HDAC1 and confirmed nuclear upregulation following combination treatment (Figure 10B). We next examined phosphorylated levels of HDAC4 (Figure 5 and Figure 7). Phosphorylation of HDAC was previously shown to repress its activity by nuclear export [68, 69]. We found a clear repression of phospho-HDAC following combination treatment for 96 h in PEO4 and OVCAR4 cell line. However, in SKOV3, we could not see such repression (Figure 10C).

### Inhibition of HDAC and DNA methyl transferases induce NRF2, HO-1 and transcriptional antioxidant response, and disrupt immunotherapy dependent repression of NRF2

While the diminished levels of NRF2 protein seen earlier could be partly explained by induction of KEAP1 during some treatments, the same does not explain its transcriptional repression. Upregulation of HDAC1 as seen in Figure 10 on the other hand warrants itself for further study, as this could be a potential mechanism of transcriptional repression of NRF2, especially upon the finding of HDAC1 induction following combination treatment.

For these reasons, we next tested this assumption by repeating exposure of our cells to the combination of Pertuzumab and Trastuzumab, but also treating cells with combination of inhibitors of HDAC1, Trichostatin (TSA) or that of DNA methylation, namely 5-azacytidine (5-Aza) [70, 71], collectively called T/A. Both these inhibitors are expected to relief transcriptional inhibition of genes that are under epigenetic regulation and silenced by DNA methylation dependent mechanisms. We found that not only did T/A lead to NRF2 induction, suggesting relief of methylation dependent suppression of its gene expression, but more importantly, it also disrupted the inhibitory action of 96 h of HER2 inhibitors treatments (Figure 11A). We could also show such T/A dependent induction of both NRF2 and its downstream transcriptional target, HO-1 at single cell level by performing immunostaining (Figure 11B). This important finding firstly demonstrated that *NRF2* gene expression could be subjected to epigenetic regulation involving DNA methylation and/or acetylation; secondly, that the inhibition of NRF2 following exposure to HER2 targeting drugs involves the above mechanism; thirdly that as such, this could be disrupted by using specific inhibitors also leading to induction of NRF2 substrates. We next made use of AREc32 cell line, the stable clone of antioxidant response reporter to further study the consequences of HDAC1 and DNA methylation inhibition on NRF2 dependent antioxidant response pathway. In this strategy, we used TSA and 5-Aza either alone or with classical activators of NRF2 to see whether that would lead to further antioxidant activation. While the classical NRF2 activator, *tert*-butylhydroquinone (tBHQ) and sulforophane induced ARE signal by 13 and 2.5 fold respectively, TSA alone led to almost 35-fold induction (Figure 11C). Furthermore, TSA could further enhance ARE signal in tBHQ treated cells to more than 70 fold. These findings further validated our earlier assumption of the involvement of epigenetic mechanisms in regulating NRF2 and hence its downstream antioxidant pathway.

To further confirm the transcriptional mechanism of NRF2 regulation following T/A treatments, we used prNRF2, our cloned *NRF2* luciferase based promoter assay as its transcriptional reporter. We found that while combination treatment of Pertuzumab and Trastuzumab repressed *NRF2* transcription, cotreatment with T/A disrupted this repression in both PEO4 and SKOV3 cell line with the latter showing a more pronounced induction of *NRF2* transcription following T/A cotreatment (Figure 11D).

Altogether, findings in this set of experiments demonstrate the involvement of transcriptional and epigenetic mechanisms of NRF2 regulation with its effects seen both at the transcriptional, single cell and overall protein levels.

### Anti HER2 targeted therapy causes NRF2 promoter methylation explaining its transcriptional repression following HER2 inhibition

**Figure 12:**
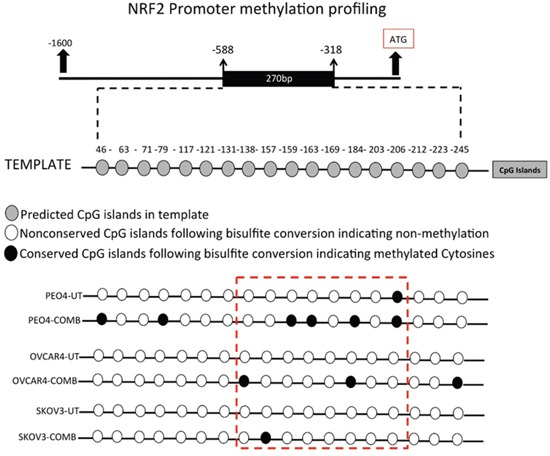
Combination of HER2 targeting immunotherapeutic agents, Pertuzumab and Trastuzumab cause hypermethylation of NRF2 promoter Exponentially growing PEO4, OVCAR4 and SKOV3 cell lines were seeded in 60mm tissue culture plates and either left untreated in media containing 1nM HRG, or treated either individually or in combination with 20μg/mL of Pertuzumab and Trastuzumab for 96 h. Following this, cells were harvested for total genomic DNA, bisulfite converted, quantified and subjected to PCR using specific primers flanking the predicted CpG methylation sites in NRF2 promoter. PCR products were sequenced, analyzed and examined for conserved CpG dinucleotides indicating methylation.

In order to more directly determine the epigenetic basis of *NRF2* regulation following combination of HER2 targeting drugs, we performed epigenetic study of *NRF2* promoter involving methylation profiling. This was also done to explain the transcriptional and protein induction of NRF2 followed by activation of its antioxidant function upon inhibition of HDAC and DNA methylation. To do this, we first performed *in silico* analysis of the *NRF2* promoter to identify CpG islands (Supplementary Figure S1A). This was done using the CpG analysis program Methprimer (http://www.urogene.org/methprimer/). This analysis identified a CpG island containing 18 CpG dinucleotides (Figure 12C and Supplementary Figure S3A). We next designed primers specific for amplification of bisulfite converted DNA but not unconverted DNA, exposed all three cell line models of this study to 96 h of Pertuzumab and Trastuzumab combination treatment, isolated total genomic DNA from exposed cells and performed its bisulfite conversion. Such conversion converted all the non-methylated cytosine nucleotides in the DNA to Thymine, whereas the methylated cytosines being resistant to such conversion remained intact. Using the designed bisulfite conversion specific primers flanking the predicted CpG island, we set up PCR, amplified this region, purified and sequenced it (Supplementary Figure S3). Careful analysis of the sequencing results revealed striking differences in the sequences between untreated and treated cell lines (Table 2). We found that only one CpG dinucleotide among the 18 predicted was methylated in the PEO4 cell line in the untreated state (CpG dinucleotide at position 206, Figure 11C). However, following treatments, we found a total of six CpG dinucleotides retained following conversion, suggesting their methylation (CpG dinucleotides at positions 43, 79, 159, 163, 184 and 206, Figure 11C). OVCAR4, which showed the least inhibition of NRF2 protein following drug treatment also exhibited limited CpG methylation with only three CpG dinucleotides showing methylation (position 138, 184 and 245). A surprising finding was with SKOV3, which only exhibited a single conservation of the CpG dinucleotide, suggesting a single methylated cytosine at position 157 (Figure 12C). The disparity between a very pronounced NRF2 protein inhibition in SKOV3 (Figure 5C) while still very limited methylation profile could be explained by the fact that KEAP1 induction during the same treatments was the highest in this cell line potentially accounting for this as a contributing factor in NRF2 repression.

**Table 2:** Conserved CpG dinucleotides demonstrating methylated cytosines

Cell Line	Conserved CpG dinucleotides			
	UT	Position	Treated	Position *
PEO4	1	206	6	46, 79, 159, 163, 184, 206
OVCAR4	0	-	3	138, 184, 206
SKOV3	0	-	1	157

* Nucleotide position within the 270bp CpG island in NRF2 promoter (see Supplementary Figure S3).

This is the first report of *NRF2* promoter methylation profiling following HER2 targeted immunotherapy. This novel finding demonstrated for the first time that the human *NRF2* promoter in three ovarian cancer cell lines exhibited methylation following combination treatment and hence identifies a novel mechanism of transcriptional repression of *NRF2*.

## DISCUSSION

The introduction of monoclonal antibody based targeted anticancer immunotherapy in the form of HER2 targeting Trastuzumab (Herceptin) and Pertuzumab has opened a new chapter in cancer immunotherapeutics. It has significantly improved our understanding of the biology of HER2-related cancers and the emergence of novel anti-HER2 drugs for the treatment of cancers. While several mechanisms, including inhibition of proliferation and angiogenesis, DNA repair and extracellular domain cleavage for recruiting host immune natural killer (NK) cells and triggering of an antibody-dependent cell-mediated cytotoxicity (ADCC) process [75] have been identified for Trastuzumab, the mechanism of action of Pertuzumab has appeared limited to primarily inhibiting signal transduction by blocking the function of HER2. Several *in vitro* and *in vivo* studies have clearly elucidated this mechanism of action by Pertuzumab [76–79]. Although these therapies work by different mechanisms, it appears that to exert an antitumor effect, they should for example inhibit phosphorylation of HER3 and antagonise the PI3K/AKT pathway [25, 28, 29]. Detailed experimental and clinical studies have shown the complimentary and enhanced efficacy and safe tolerability of HER2 targeting and blockade by the novel combination of Trastuzumab with Pertuzumab [32, 80–84]. However, treatment outcomes with single agent or combination of agents remain fairly unpredictable, tumour type specific and tumour biology dependent, especially the expression levels of cell surface receptors, their dimerization preferences, recycling kinetics and ligand abundance [25, 33–35, 85]. Also the molecular mechanisms responsible for susceptibility and *de novo* and/or acquired resistance to these HER2 targeted immunotherapeutic agents are poorly understood. In this study we aimed to identify and characterise novel signalling pathways that might explain the efficacy of HER2-targeted immunotherapies that are critical to avoid or to overcome resistance.

In the current study, we sought to determine the mechanism of action of targeted immunotherapeutic agents and in particular to understand the enhanced cytotoxic response triggered uniquely by combination of immunotherapeutics rather than individual agents. We determined the degree of sensitivity of ovarian cancer cells derived from different origins to the HER2 targeting antibodies, Pertuzumab and Trastuzumab, either employed alone or in combination. These ovarian cancer cell lines are of low (OVCAR4), moderate (PEO4), and high (SKOV3) HER2 expression status [34], in addition to having variable expression levels of HER3, the key dimerization partner of HER2 in the order PEO4>OVCAR4>SKOV3 [34]., We found that at least part of mechanism of action of the HER2 targeted immunotherapeutic agents involved generation of ROS, which contributed to the killing effects and cancer growth retardation. This is consistent with the conventional adage that depletion of GSH can cause oxidative stress and sensitise tumours to the killing effects of the therapeutic agents. These observations were further reinforced by the use of NAC to attenuate ROS and desensitise, or of RA to elevate ROS, and augment the cytotoxicity of Trastuzumab and Pertuzumab.

The cell lines exhibited complex and different degrees of cytotoxicity to Pertuzumab and Trastuzumab alone or their combination. However, the combination treatments produced the greatest cytotoxicity and killing effect, as well as the highest inhibition of RTK signalling, consistent with previous observations [25, 32, 33]. Since we have recently characterised these cell lines as having different levels of NRF2, KEAP1, hierarchical addiction to ROS and its potential for manipulation, intricate with their proliferative capacity [10, 11], we postulated any or all of these to be possibilities for the observed differential response to the different immunotherapies, especially the combination therapy. The rationale for this postulate was the fact that RTK and AR pathways share common substrates, both pathways are cytoprotective and pro-survival in nature and both these have been implicated in drug resistance. Additionally, recent studies have explored and identified co-modulatory and co-regulatory roles of the two pathways [36–39].

Interestingly, combination of the targeted immunotherapeutic agents (Pertuzumab and Trastuzumab) caused down regulation of NRF2, induction of KEAP1, and depletion of glutathione, features that were ascribable to inhibition of the NRF2 dependent antioxidant transcriptional program. These demonstrated that HER2 targeting monoclonal antibodies repress the NRF2 dependent antioxidant pathway, which may contribute to the enhanced cytotoxicity for the combination of Pertuzumab and Trastuzumab.

Next, we sought evidence that the mechanism of action and effectiveness of HER2 targeted immunotherapies, in particular the combination of Trastuzumab and Pertuzumab, *in vivo*, is characterised by inhibition of NRF2 function. We did this by performing a series of *in vitro* experimentation, as well as bioinformatic analysis of microarray data from the *in vivo* SKOV3 xenograft model of HER2 targeting immunotherapies [32] as before [25]. In our analysis of gene expression we focused on the NRF2 network using a knowledge-based approach that was informed by previous reports [50–54]. Firstly, we demonstrated that the suppressive effects of combination therapy on the AR pathway seen *in vitro* (Figure 1 and Figure 2) were reproduced *in vivo* (Figure 6–8). Significant changes in the gene expression of NRF2 and the NRF2 network signalling were observed following treatment with anti-HER2 immunotherapies in the SKOV3 xenograft. The most dynamic effects and changes were seen with the combination therapy. Up regulation of some of the genes within the NRF2 network can be explained by the takeover of transcriptional control of such genes by other significantly induced transcription factors like the FOXO family and NFE2L1 (NRF1). Thus the *in vivo* model supported and confirmed the results of our *in vitro* model, which showed that the effectiveness of HER2-target immunotherapy is greatly and significantly informed by repression of NRF2 and its antioxidant function. A number of classical NRF2-dependent genes like *AKR1C1, ATP1B1, BRCA1, BRCA2, GAPDH, GCLM, GCSH, GSTM4, H6PD, HMOX1, IDH1, NQO1, PRDX1, SOAT1, SOD2, UGDH TUBB3*, and *VEGFA* were suppressed, especially with combination therapy.

Several possible mechanisms of NRF2 inhibition seen *in vitro* and *in vivo* could be identified and explained from the microarray analyses. First, the classical NRF2 and KEAP1 relationship, levels and dynamics appeared to be in place following the HER2 target immunotherapies *in vivo* as observed with the *in vitro* model. The expression of *NRF2* significantly decreased following combination treatment. This supports the notion that NRF2 opposes the action and effectiveness of Trastuzumab and Pertuzumab, perhaps by promoting the sequestration of the ROS generated to kill cells following immunogenic drugs administration. Further, this notion is strengthened and supported by the observation that the expression of *KEAP1* was significantly induced following both single and combination treatments with least induction observed under combination treatments. However, the ratio of *NRF2*/*KEAP1* expression was higher under single agent than with combination treatments, suggesting greater degradation and inhibition of NRF2 function [7, 8] with combination than with single agents.

The mechanisms by which retinoic acid (RA) inhibits NRF2 and its function are known [62, 86] and have been the basis for our use of RA to modulate cellular NRF2 status, ARE-dependent transcriptional program and to implicate NRF2 in regulating cellular susceptibility to HER2 targeted immunotherapies. Interestingly, we found increased expression of *RXR* and *RARA* genes following Trastuzumab, Pertuzumab, and their combination treatment of the SKOV3 xenograft tumours. This suggested that these mechanisms may play a part *in vivo* to inhibit NRF2 function and thus influence the effectiveness of the HER2 target immunotherapies. Also *GSK3* expression appeared stable and the expression of *FYN* kinase was induced, by all the immunotherapies against the SKOV3 xenograft tumours, especially with combination therapy where the expression of *β-TrCP* was induced, which highlights another possible mechanism of controlling NRF2 levels and functions [87–89]. Further, the down and up regulation of *BACH1* and *BACH2* expression following single agent and combination therapy, respectively, could help explain the greater loss of NRF2 function in the combination therapy, since BACH is a negative regulator of ARE-NRF2-dependent transcription of genes [90–92]. Moreover, there was up regulation of expression of *MAF* gene family (*MAFF, MAFG, MAFK*), especially following combination therapy, an observation that agrees with the notion of the negative regulation of ARE-NRF2 dependent transcriptional program [93, 94].

Further analyses and visualisation of the *in vivo* gene expression data confirmed perturbations in the NRF2 network and down regulation of some NRF2 target genes, especially genes associated with antioxidant responses (*AKR1B1, AKR1C1, HMOX1, NQO1, FRMD6, GAPDH, IDH1, PRDX1, SOAT1, SOD2*) and glutathione metabolism (*ATP1B1, ATP2A1, GCLM, GCSH, GSTM4, H6PD*). These supported the role of ROS production and the undermining of NRF2 status and functions as the bases of action and effectiveness of the immunotherapies, especially with the combination of Trastuzumab and Pertuzumab. It is pertinent to describe the up regulation of expression of *HDAC*s (*HDAC1, HDAC3, HDAC5, SIRT7*), histone methyl transferases with CpG binding protein (*CXXC1*) and certain nuclear co-repressor genes (*NCOA6, NCOR1, PRC1, RCOR3*), as well as the down regulation of expression *HAT* and its related functional homologues or orthologues like *DNA2, HES1, MED4, MET, METRN, METTL1*. These observations highlighted genetic and transcriptional packaging, suturing and control, which consequently led us to hypothesise that transcriptional silencing and epigenetics, as shown before [95, 96] may contribute to the distinct mechanisms of inhibition of NRF2 function by combination immunotherapy. These data and those from the *NRF2* gene promoter methylation, transcriptional assays and HDACs dynamics using our *in vitro* cell models supported the hypothesis. Overall there is agreement between the *in vitro* and the *in vivo* data which together illustrate the important role of NRF2 in influencing outcomes to targeted therapies involving HER2 receptor inhibition. Moreover, our recent work on the regulation of HER2 and HER3 by NRF2 to oppose HER2 targeted immunotherapy [45], gives further support and credence to this assertion. The study has also opened up a new potential avenue of improving the effectiveness of Trastuzumab (Herceptin), which currently benefits less than 30% of breast cancer bearing patients and as Trastuzumab-associated chemotherapy can modulate the pro-inflammatory markers of HER2-positive breast cancer patients [97].

## CONCLUSION

We have elucidated a novel mechanism of action for the combination of HER2 target anticancer immunotherapeutic agents. We demonstrate that combination treatment with HER2 targeting monoclonal antibodies Pertuzumab and Trastuzumab cause greater inhibition of NRF2 function and subsequent greater repression of NRF2 dependent antioxidant responses in human ovarian cancer cell lines. The degree of repression of NRF2 determines the overall sensitivity of cancer cells towards the HER2 targeted therapies, an axis that could be modulated to further sensitise otherwise resistant ovarian cancer cells. Furthermore, we present evidence that methylation leading to transcriptional repression and gene silencing at the *NRF2* promoter occurs following combination of Pertuzumab and Trastuzumab treatments. Therefore the greater effectiveness and enhanced cytotoxic action of the combination of the HER2 targeted immunotherapeutic agents may be at least partially explained by their unique ability to cause transcriptional inhibition of NRF2 and greater repression of its antioxidant function in low, moderate and high HER2 expressing ovarian cancer cell lines. This study expands the role of NRF2 as a key element in driving drug resistance and opens up a novel strategy of sensitising cancer cells to HER2 targeted therapy, as well as overcoming the resistance of cancer cells to such immunotherapeutics.

## MATERIALS AND METHODS

### Cell lines, culture conditions and treatments

Human ovarian cancer cell lines PEO4, OVCAR4 and SKOV3 were maintained in RPMI 1640 media (Gibco® Invitrogen) supplemented with 10% foetal bovine serum (FBS), 2 mM glutamine, 1 mM sodium pyruvate, 100 μg/ml streptomycin and 100 U/ml penicillin in an atmosphere of 5% CO_2_ and incubated at 37°C. Before experimental treatments, cells were grown for 24 h in RPMI 1640 media prepared as above but replacing FBS with 5% double charcoal stripped FBS (Fisher). Heregulin-β1 (HRG, Sigma) was used by preparing 1 μM stock solution made with 5% trehalose, 10% FBS in PBS and diluted to a final concentration of 1nM with media during treatments. Monoclonal antibodies targeting HER2 receptor, Pertuzumab and Trastuzumab or their combinations were used by directly diluting the drugs in media to a final concentration of 20 μg/mL. For Retinoic acid (RA) treatments, a stock solution of 40 mM was made in 100% ethanol in amber Eppendorf tubes pre-aired with nitrogen gas. Once the stock solution was made, it was bubbled again with nitrogen gas and closed, stored at −80°C and protected from light until further use. A final concentration of 2.5 μM was used for treatments. For reducing conditions, 100 mM N-Acetyl Cysteine (NAC, Sigma-Aldrich) was prepared in deionised water and diluted to a final concentration of 10 mM with media during treatments. For ROS detection, 2′,7′-Dichlorofluorescin diacetate(DCFDA, Sigma) solution was prepared with Dimethylsulfoxide in amber tubes to a concentration of 50 mM and stored at −20°C in dark until used. For cytotoxicity assay, 3-(4,5-Dimethylthiazol-2-yl)-2,5-Diphenyltetrazolium Bromide (MTT) was used by making a stock solution of 5 mg/mL in PBS and filter sterilising it. The solution was stored at 4°C in the dark until used. To inhibit DNA methylation, 5-azacytidine and HDAC inhibitor Trichostatin A (collectively referred to as T/A) were used at final concentration of 2 μM and 200 nM respectively. Mock treatments with DMSO were performed in parallel.

### Reactive oxygen species (ROS) detection

The ROS detection assay was performed by using 2′,7′-Dichlorofluorescin diacetate (DCFDA) staining (Sigma). Briefly, cells were seeded in triplicate at a density of 5×10^3^ cells/well in opaque flat bottom 96-well tissue culture plates in 100μl media without phenol red and allowed to grow for 24 h before being exposed to different treatments. Following required treatments, a 50mM stock solution of DCFDA was added to each well containing 100 μL pre-existing media to achieve a final concentration of 25 μM and incubated for 45 min at 37°C. Fluorescence signal intensities indicating ROS levels were recorded by taking readings using 96-well fluorescent multi plate reader (MODULUS™, Promega) using excitation and emission spectra of 485/535 nm. To normalise the fluorescence signal, cells in the same wells were subsequently stained with coomassie brilliant blue stain (Sigma-Aldrich) for 1 h, washed with distilled water and 10% SDS solution added to release the absorbed dye for 10 min while shaking. The absorbance values at 595 nm were then recorded using multiplate absorbance reader (MODULUS™, Promega) and data used to normalise the fluorescence values.

### Protein extraction and immunoblotting

For immunoblotting, cells were seeded in 60mm tissue culture plates and grown until 70% confluent and exposed to required treatments. At protein harvest, cells were trypsinized (Gibco® Invitrogen), washed with PBS, and harvested to obtain protein lysates using RIPA buffer (Pierce Biotech) supplemented with protease and phosphatase inhibitor cocktail (Pierce Biotech). The lysates were subjected to sonication of 2 cycles for 10 s at 50% pulse. The final mixture was shaken gently on ice for 15 min and the protein supernatant was obtained following centrifugation of the lysates at 14000 x g for 15 min. Proteins obtained were quantified by Bradford assay (Sigma-Aldrich) using BSA as a standard and sample buffer (Nupage LDS, Invitrogen) was added to protein lysates, heated at 70°C for 20 min and stored at −20°C until further use. On the day of immunoblotting, lysates were loaded into wells of 4-12% gradient SDS-polyacrylamide gels (Nupage® Bis-Tris gels, Life Technologies) and subjected to electrophoresis at 200V for 1-2 h. Following this, proteins were transferred to polyvinylidene difluoride membranes (GE Amersham) using the XCell SureLock Mini-Cell system (Invitrogen) at 50V for 90 min and processed using a commercially available kit (WesternBreeze^™^ Chromogenic Immunodetection Kit, Invitrogen). Non-specific reactivity was blocked by incubation with the blocking reagent supplied in the kit. Membranes were further treated by incubating with primary antibodies (Table 1) for 2 h at room temperature or overnight at 4°C, followed by incubation for 30 min at room temperature with appropriate secondary anti rabbit antibody supplied in the kit. Bands were visualized with the BCIP/NBT based chromogenic substrate. For loading control, either immunoblotting of the same lysates was performed using β-Actin antibody (Abcam Bioscience, UK) or the PVDF membranes with transferred proteins visualized using Ponceau stain (Sigma).

**Table 1:** Antibodies used in the study

Antibody	Host	Catalogue Number	Company
NRF2	Rabbit	Sc-722	Santa Cruz
KEAP1	Rabbit	4678S	Cell signalling
Phospho Her2 T877	Rabbit	2241S	Cell signalling
Phospho AKT 473	Rabbit	4060S	Cell signalling
HDAC1	Mouse	5356	Cell signalling
PhosphoHDAC	Rabbit	3443	Cell signalling
Heme Oxygenase 1 (HO-1)	Rabbit	sc-10789	Santacruz
Alexa fluor 488 conjugated secondary antibody	Rabbit	ab150077	Abcam
Alexa fluor 568 conjugated secondary antibody	Rabbit	ab175471	Abcam
β-actin	Rabbit	ab1801	Abcam

### Cloning and expression vector

The 1.5kb proximal promoter region of *NRF2* upstream of its translational start site was cloned into a luciferase reporter vector (Promega) and used to study the transcriptional regulation of *NRF2*. Table 3 lists the primers for the amplification of the *NRF2* promoter. Total genomic DNA was isolated from human cells using DNeasy Blood and tissue kit (Qiagen) and quantified using AstraGene microvolume spectrophotometer (AstraNet). 100ng of the genomic DNA was used to amplify the promoter sequences with MyFi mix (Bioline), using primers that incorporated *XhoI* and *NcoI* restriction endonuclease sites 5′ and 3′ ends of the amplified *NRF2* promoter respectively. PCR conditions for promoter amplification were initial denaturation of 95°C for 7 min followed by 35 cycles of 95°C for 30 s for denaturation, a gradient annealing temperature of 50^−^60°C for 30 s and 72°C for 90 s for extension and a final extension for 10 min at 72°C. The PCR products were subjected to electrophoresis and extracted from agarose gel (Qiagen), digested using *XhoI* and *NcoI* restriction enzymes (Promega) and ligated into pGL3 basic luciferase vector (Promega) to create the *NRF2* promoter construct (prNRF2) driving the expression of Luciferase gene for utilization in a Dual luciferase reporter assay (Promega). The integrity of cloned sequences was determined by commercial sequencing service (www.dnaseq.ac.uk). The *NRF2* promoter construct was transfected into relevant cell lines using Lipofectamine® 3000 (Life Technologies) as a transfection reagent.

**Table 3:** Primer sequences

Primers	Sequence
Biseq forward	5- TGAGATAAAAGTAGGGTAAGGTTTTGTA-3
Biseq reverse	5- ACTACCAACTAAAATCCCAACAAAC-3
prNRF2 forward	5- CTC GAG GGC GTT GAT TGC TAT AGT CAG G-3
prNRF2 reverse	5- CCA TGG GAT GAG CTG TGG ACC GTG TG-3
GCLC F forward	5-TCTCTAATAAAGAGATGAGCAACATGC-3
GCLC R reverse	5-TTGACGATAGATAAAGAGATCTACGAA-3
Probe	5FAM-CAGGAGATGATCAATGCCTTCCTGCAAC-3BHQ

### Luciferase reporter assay

For the analysis of promoter activities and transcriptional regulation of *NRF2*, the 1.5 kb promoter region of *NRF2* gene cloned in pGL3 basic vector (Promega) was transfected into relevant cell lines. Briefly, cells were seeded in triplicates in 24-well plates at a density of 2 × 10^5^cells per well and allowed to attach for 24 h. Following this, cells were either transfected with 1 μg of empty pGL3 basic vector (Promega) or PGL3 basic vector with cloned fragments of *NRF2* promoter driving the expression of luciferase gene, using transfection reagent Lipofectamine 3000^®^ according to manufacturer's instructions (Life Technologies). Co-transfection was also performed with 0.2 μg of pRL-CMV vector (Promega) expressing renilla luciferase to provide an internal control of transfection. Following this, cells were allowed to grow for 24 h, subjected to desired treatments, lysed and protein lysates transferred to opaque white bottom 96-well plates. The dual luciferase activity of fire fly luciferase (from cloned promoters) and Renilla (internal control) in the harvested lysates was measured sequentially by following manufacturer's instructions (Promega) and taking luminescence readings in a luminometer (MODULUS™, Promega). To determine the NRF2 dependent transcriptional antioxidant response following different treatments, stable clones of MCF7 cells carrying pGL3 vector with a cloned 8 copies of *Cis*-Antioxidant Response Elements (ARE) were used as stable luciferase reporter cells (AREc32) to report NRF2 dependent transcription [98]. Briefly, AREc32 cells were seeded in quadruplicate in 24-well plates at a density of 0.5 × 10^5^ cells per well and allowed to attach for 24 h. Next day, cells were washed with pre-warmed PBS, treated as required and further allowed to incubate for the desired time period. Towards the end of treatments, 250 μL of the reconstituted luciferase reagent (Bright Glo™ Luciferase, Promega) was added in each well containing 250 μL of pre-existing media and the plate incubated at 37°C for 10 min. 100 μL of the cell lysate was transferred to opaque white bottom 96-well plate for luminescence detection in a luminometer (MODULUS™, Promega) while 50μL was subjected to Bradford assay to estimate protein content for the normalization of luminescence signal.

### Cytotoxicity assay

Cytotoxicity assays were performed using 3-(4,5-Dimethylthiazol-2-yl)-2,5-Diphenyltetrazolium Bromide (MTT). Briefly, cells were seeded in triplicates at a density of 0.5 × 10^4^cells in 96-well plate and allowed to attach for 24 h. Following this, old media was removed and 80 μL of media containing relevant drugs was added and the plate incubated for the required period of time. On the day of assay, 20 μL of the 5mg/mL MTT stock was added to each well and the plate further incubated for 4 h. Following this, the old media with MTT was removed, cells gently washed with pre-warmed PBS and 100 μL of DMSO added to solubilize the internalized MTT by shaking the plate over an orbital shaker for 15 min. Absorbance of the released dye was measured and recorded using multiplate reader (MODULUS^™^, Promega) at 540 nm.

### Immunocytochemistry/immunolabelling

For immunocytochemistry, exponentially growing cells were seeded at a density of 5 × 10^4^ cells in media onto poly-L lysine (Sigma-Aldrich) coated cover slips placed in a 12-well tissue culture plates and allowed to attach for 24 h. Following relevant treatments, cells were washed three times with ice cold PBS and fixed in 3.5% paraformaldehyde in standard PBS at room temperature for 30 min. Next, cells were gently washed twice with 1 ml of PBS, permeabilized with 0.3% triton X-100 in PBS for 10min, and following three washes with PBS, blocked with a solution containing 1% goat serum, 1% bovine serum albumin and 0.05% Triton X-100 in PBS for 30 min. Cells were then incubated with relevant primary antibody (Table 1) diluted in blocking solution for 1 h, washed three times with 0.1% Triton X-100/PBS for 5 min, and then incubated with Alexa Fluor 488 or 568- conjugated goat anti-rabbit secondary antibody (Table 1) for 30 min. After subsequent three washes with the 0.1% Triton X-100 in PBS for 5 min, cover slips with cells were mounted on slide using 4′,6-Diamidino-2-Phenylindole, Dihydrochloride (DAPI)-containing mounting reagent (Life technologies) and imaged under relevant filters with a Leica DMiRe2 electronic microscope.

### Imaging and analysis

Quantitative analysis of raw immunoblots was performed by capturing the images in high resolution TIFF format files using a charge-coupled-device camera (AxioCam MRc, Carl Zeiss) and subjected to Gelpro analysis software, version 3.1 (Gelpro Media Cybernetics) for integrated optimal densitometry. Fluorescence images of immunocytochemistry were collected under relevant excitation and emission filters depending on the fluorotype under Leica DMiRe2 electronic microscope equipped with iXonEM +897 EMCCD camera (ANDOR Technologies Ltd). Images were analyzed using multidimensional microscopy software Andor Module iQ Core. Colocalization assay was performed and determined with software integral features supplied by Andor IQ core software. Data were generally expressed as mean ± S.D. for individual sets of experiments.

### Bisulfite sequencing and promoter methylation analysis

Bisulfite conversion of the genomic DNA extracted from ovarian cell lines was performed using EpiTect Fast LyseAll Bisulfite Kit (Qiagen) using manufacturer's instructions. Briefly, ovarian cancer cells were seeded in 60 mm plates and allowed to attach for 24 h. Following this, cells were exposed to different treatments for required period of time, trypsinized, centrifuged and pellets suspended in 150 μL cold PBS. From this mixture, 10 μL was used for DNA extraction using the lysis buffer supplied in the kit, bisulfite converted following manufacturer's instructions and the final elute quantified using AstraGene microvolume spectrophotometer (AstraNet) to obtain a concentration of 20 ng/μL genomic DNA. Next, 100 ng of the extracted DNA was subjected to PCR amplification (Myfi mix, Bioline), using bisulfite specific primers (Table 3) applying PCR conditions of 95°C for 7 min for initial denaturation followed by 30 cycles of 95°C for 30 s denaturation, 50°C for 30 s for annealing and 72°C for 90 s for extension and a final extension at 72°C for 10 min. Unconverted DNA extracted from same cells were used as negative controls. The products were run on 1.5% agarose gel and analyzed for integrity and size (Supplementary Figure S3) and sent to a commercial sequencing service (https://www.dnaseq.co.uk) to identity the sequences. For *in silico* analysis of promoter methylation and prediction of CpG islands in *NRF2* promoter, the Methprimer (www.urogene.org) online resource was used. Firstly, a 1.5 kb human *NRF2* promoter sequence was retrieved from Ensembl genome browser (Ensembl.org) and provided as input to the Methprimer software. Following further analysis, a 270 bp region with 18 predicted potential methylated sites was identified. The primers, which were specific for Bisulfite converted DNA, were selected and ordered (Table 3) and used to amplify 270 bp region of genomic DNA isolated from PEO4, OVCAR4 and SKOV3 cells following relevant treatments.

### Quantitative real-time PCR (qRT PCR)

Quantitative real-time PCR (qRT PCR) was performed using Taqman^®^ Universal master mix (Life technologies). The probes and primers for human *NRF2* (Hs00975961_g1), *HO-1* (Hs00157965_m1) and 18s RNA (Hs03928992_g1) were obtained from life technologies and previously validated by the manufacturers. For *GCLM*, custom-made primers were used with the sequence and probe listed in Table 3. Real time PCR, analysis and processing of obtained data were performed by Stratagene Mx 3000P PCR amplification machine and built-in software (Agilent Technologies). Briefly, PEO4, OVCAR4 and SKOV3 cell lines were seeded in 60 mm plates and grown until 70% confluent. Following this, cells were washed once with warm PBS and new media with required treatments added for the desired period of time. Total RNA was extracted from treated cells using Trisure (Bioline) and its integrity and quality was determined by agarose gel electrophoresis and quantified using AstraGene microvolume spectrophotometer (AstraNet). 500 ng RNA was next used to convert to cDNA with the Affinity Script cDNA synthesis kit (Agilent Technologies). The cDNA was subjected to qRT PCR using relevant probes and primers as listed in Table 3.

### Statistical analysis

All statistical analysis were performed using statistical software SPSS (IBM, version 22). Test for normality of data was determined by Shapiro-Wilk and Kolmogorov and Smirnov tests. The significance (p value) of differences of pooled results was determined by either independent t tests or One WAY ANOVA followed by post hoc Tukey's tests. Significance was defined as * = p< 0.05, ** = p<0.01, *** = p<0.001.

### Glutathione assay

Total cellular Glutathione (GSH) levels were determined by using the GSH assay kit (Sigma) according to the protocols described by Tietze F, 1969 [99].

### Bioinformatics methods and *in vivo* analysis

Microarray data on gene expression (GSE31432; NCBI Gene Expression Omnibus https://www.ncbi.nlm.nih.gov/geo) following Trastuzumab (20 mg/kg), Pertuzumab (20 mg/kg) and the combination treatment from SKOV3 tumor xenografts in mice were used [32, 100]. In this paper, we focused on gene expression relating to the NRF2 network and downstream targets, as well as the glutathione and epigenetics signatures, which are modulated by the anti-HER2 therapies. Different visualization methods were used to represent the results of statistical analysis of expression data. In addition to typical clustering of expression data in the form of heatmaps, we generated and analyzed volcano plots, Venn diagrams and signaling network maps. Volcano plots represent fold change in gene expression level together with the statistical significance of this change [32, 100, 101]. Signaling network maps combine gene expression data with protein signaling network downloaded from the KEGG database (http://www.genome.jp/kegg/), the work of Papp D et al, 2012 [102] and other knowledge-based approach and sources [103–106]. We used the R programming language for statistical processing of the data (Student's *t*-test) with the Bioconductor package for processing the data and Cytoscape (http://www.cytoscape.org) with CytoKEGG plugin for network illustration to integrate the gene expression data with the KEGG signaling networks and also the NRF2-related interactome and regulome [102], downloaded and imported into the network model to Cytoscape. From there, the list of genes associated with the pathways were extracted and used in R to obtain the required gene expression values for further analysis. The means of the expression values in drug and control samples were calculated and followed by a Student's t-test to calculate the p-values for the fold changes. We then generated the heatmaps showing the log2 fold-change that the drugs caused to expression of the genes. We also used the fold-change data and the p-values to create volcano plots for the different drugs treatments using all the genes in the dataset and only for the genes associated with the different pathways examined and reported. Lastly, the ratios were exported to Cytoscape to color the nodes in the network visualization.

## SUPPLEMENTARY FIGURES AND TABLES







## Abbreviations

5-Aza: 5-azacytidine
AR: Antioxidant Response
ARE: Antioxidant Response Element
DCFDA: 2′,7′-Dichlorofluorescin diacetate
FBS: Foetal bovine serum
GSH: Glutathione
HDAC: Histone deacetylase
HER: Human epidermal growth factor receptor
HRG: Heregulin-β1
KEAP1: Kelch-like ECH-associated protein 1
MTT: 3-(4,5-Dimethylthiazol-2-yl)-2,5-Diphenyltetrazolium Bromide
NAC: N-Acetyl Cysteine
NRF2: Nuclear erythroid related factor-2
RA: All-trans Retinoic acid
ROS: Reactive oxygen species
RTK: Receptor tyrosine kinase
TSA: Trichostatin.
