# Supplementary material for: A novel mechanism of action of HER2 targeted immunotherapy is explained by inhibition of NRF2 function in ovarian cancer cells
Source: Oncotarget. 2016 Oct 4;7(46):75874–901. doi: 10.18632/oncotarget.12425 (PMC5342785; doi:10.18632/oncotarget.12425)
Supplement: Supplementary file 1 [file oncotarget-07-75874-s001.pdf]

A novel mechanism of action of HER2 targeted immunotherapy is explained by inhibition of NRF2 function in ovarian cancer cells

SUPPLEMENTARY FIGURES AND TABLES

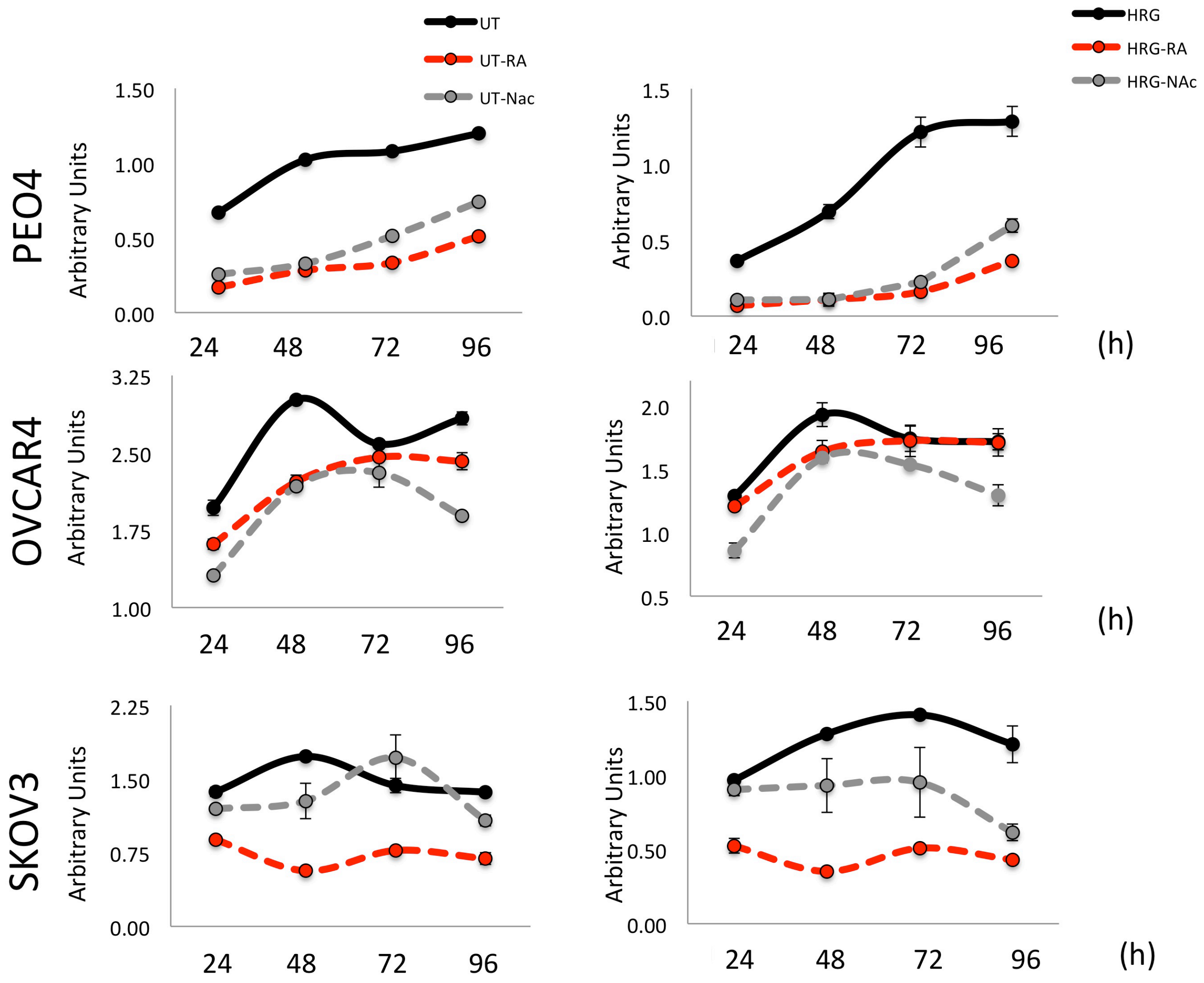

Supplementary Figure S1: Proliferation of ovarian cancer cell lines in absence or presence of either NAC or RA in growth media. Exponentially growing cells were seeded in 96 well plates in triplicates and allowed to attach for 24 h. Following this, cells were either left untreated (UT) in media containing 5% charcoal stripped FBS, or treated with same media containing 1nM Heregulin (HRG) or with cotreatments of 10mM NAC or 2.5µM RA individually for the indicated time points. MTT reagent was directly added at the end of treatments and cells further incubated for 4 h. The up taken dye was released by adding 100µL of DMSO for 15min and recording absorbance at 540nm using multiplate reader (MODULUS™, Promega). Values are means with ± S.D of triplicates and expressed as fold decrease to UT alone.

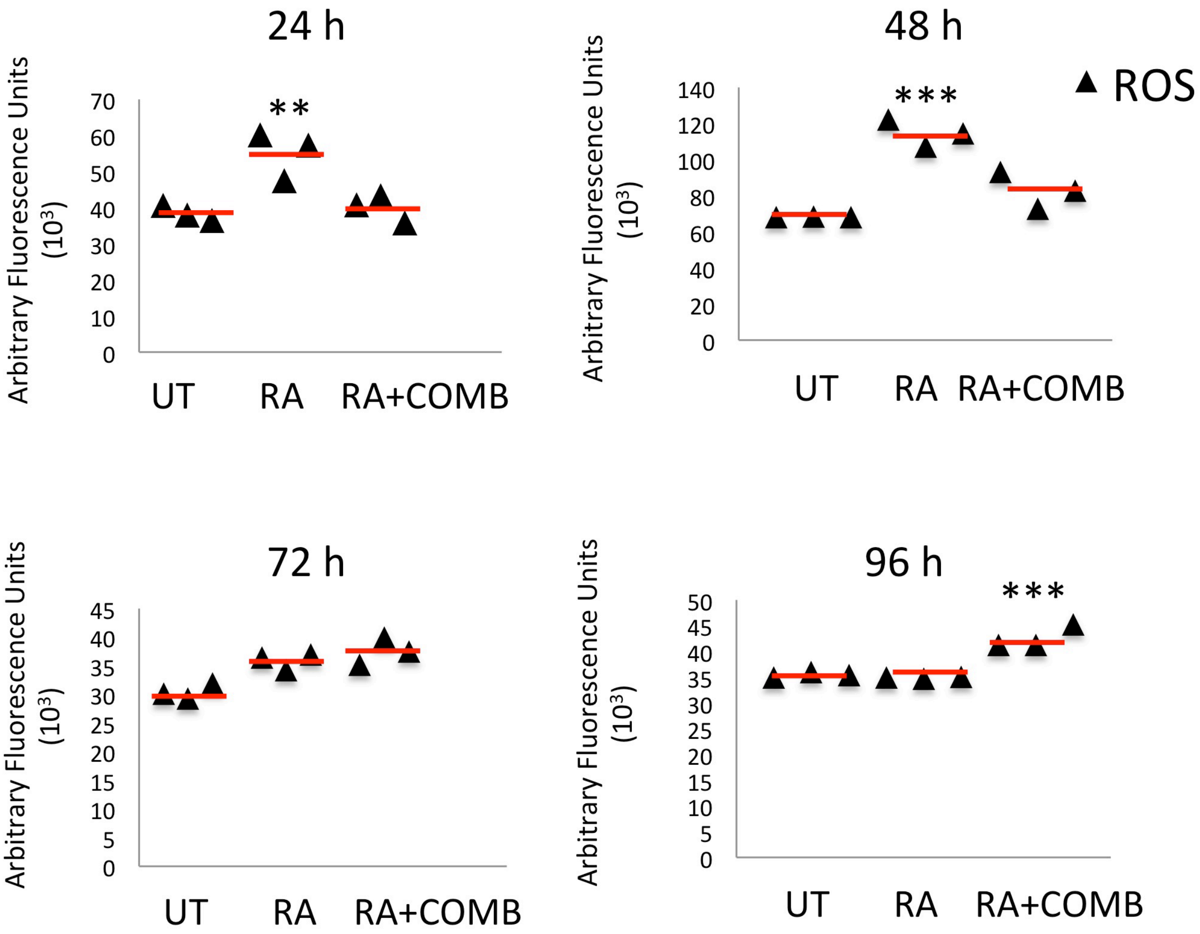

Supplementary Figure S2: Treatment with Retinoic acid (RA) causes elevation of Reactive Oxygen Species. Exponentially growing cells were seeded in triplicates in opaque flat bottom black walled 96-well plates for 24 h. Following this, cells were either left untreated in media containing 1nM Heregulin (UT) or exposed to 2.5µM RA, or cotreated with 2.5µM RA and 20µg/mL of Pertuzumab and Trastuzumab for the indicated time points. Following incubations, cells were loaded with DCFDA fluorescent stain for 45 min and assayed for ROS by measuring fluorescence using fluorescence multiplate reader (MODULUS™, Promega) with excitation and emission spectra of 485nm/535nm. Data are the means with ± S.D of triplicates and expressed as fold change with statistical significance determined by ONE WAY ANOVA followed by Tukey's post hoc test according to the scale * P <0.05, **P <0.01, ***P <0.001.

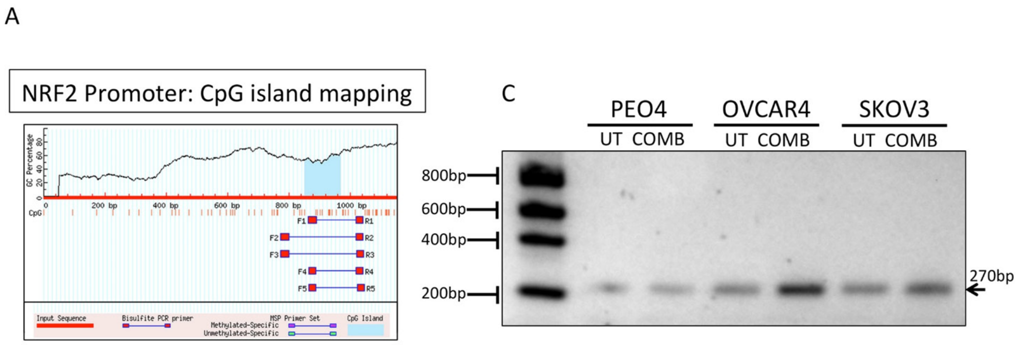

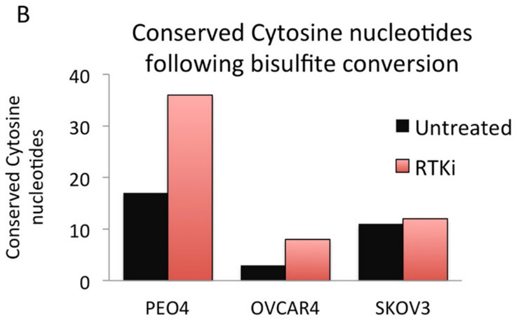

Table S1: Total conserved Cytosines

Cell Lines	UT	COMB
PEO4	17	36
OVCAR4	3	8
SKOV3	11	12

Supplementary Figure S3: promoter methylation profiling. (A) Prediction of methylated CpG dinucleotides in NRF2 promoter. Methprimer (www.urogene.org) was used to analyses 1.5kb NRF2 promoter region for potential CpG islands and methylation prediction. A 270pb stretch of promoter containing 18 CpG dinucleotides was identified for subsequent methylation analysis. **(B** and **C)** Agarose gel image of the PCR products from bisulfite converted DNA using bisulfite conversion specific primers. Total genomic DNA was extract from PEO4, OVCAR4 and SKOV3 cells previously either untreated (UT) or exposed to HER2 inhibitors (RTKi) for 96 h. The extracted DNA was quantified, bisulfite converted and subjected to PCR amplification using primers specific to the bisulfite converted DNA and flanking the 270pb region of NRF2 promoter containing the identified 18 CpG dinucleotides.

Supplementary Table S1: Identified common and differentially expressed genes associated with the NRF2 network following immunotherapies on SKOV3 xenograft tumours

See Supplementary File 1

Supplementary Table S2: Significantly down-regulated and up-regulated genes within the NRF2 network genes following the different immunotherapies ($p \leq 0.05$)

See Supplementary File 2
